# Pyroptosis-Related Signature Predicts the Progression of Ulcerative Colitis and Colitis-Associated Colorectal Cancer as well as the Anti-TNF Therapeutic Response

**DOI:** 10.1155/2023/7040113

**Published:** 2023-01-27

**Authors:** Yumei Ning, Kun Lin, Jun Fang, Xiaojia Chen, Xinyi Hu, Lan Liu, Qiu Zhao, Haizhou Wang, Fan Wang

**Affiliations:** ^1^Department of Gastroenterology, Zhongnan Hospital of Wuhan University, Wuhan, China; ^2^Hubei Clinical Center and Key Lab of Intestinal and Colorectal Diseases, Wuhan, China; ^3^Renmin Hospital of Huangmei County, Huanggang, China

## Abstract

Ulcerative colitis (UC) is a complex intestinal inflammation with an increasing risk of colitis-associated colorectal cancer (CAC). However, the pathogenesis is still unclear between active UC and inactive UC. Recently, it has been reported that pyroptosis-related genes (PRGs) are closely associated with inflammatory disease activity. Nevertheless, the specific roles of PRGs in the progression and treatment of UC and CAC remain unclear. In this study, we identified 30 differentially expressed PRGs based on the immune landscape of active and inactive UC samples. Meanwhile, weighted gene coexpression network analysis was applied to explore important genes associated with active UC. By intersecting with the differentially expressed PRGs, CASP5, GBP1, GZMB, IL1B, and IRF1 were selected as key PRGs to construct a pyroptosis-related signature (PR-signature). Then, logistic regression analysis was performed to validate the PR-signature and establish a pyroptosis-related score (PR-Score). We demonstrated that PR-Score had a powerful ability to distinguish active UC from inactive UC in multiple datasets. Besides, PR-Score was positively correlated with immune cell infiltration and inflammatory microenvironment in UC. Lower PR-Score was associated with a better response to anti-TNF therapy for patients with UC. Additionally, high-PR-Score was found to suppress CAC and improve the survival outcomes of patients with colorectal cancer. Finally, the levels of the PR-signature genes were validated both in vitro and in vivo. These findings can improve our understanding of PRGs in UC and provide new markers for predicting the occurrence of active UC or CAC and the treatment of UC.

## 1. Introduction

Ulcerative colitis (UC), a subcategory of inflammatory bowel disease (IBD), is a complex, chronic, and recurrent inflammation [1], with an increasing risk of colitis-associated colorectal cancer (CAC) occurring after 8 to 10 years of disease [2]. UC can be further divided into inactive and active states, the latter of which is known to be associated with a stronger immune response. However, the differences of immune cell infiltration between active UC and inactive UC remain unclear, and recent studies have pointed out that there are significant differences between UC and normal mucosa in this regard [3, 4]. Currently, antitumor necrosis factor (TNF) therapy is the first-line treatment for patients with moderate to severe UC [5]. Nevertheless, only two-third of the patients respond to the treatment [6]. Genetic markers are urgently needed to predict individual response to anti-TNF therapy.

Pyroptosis, an inflammatory form of programmed cell death, plays an important role in both tissue homeostasis and immune response [7]. The gasdermin (GSDM) family proteins serve as the main mediators of pyroptosis, which can be activated by caspase (CASP) family (CASP1/4/5/11) via proteolysis, and induce the formation of GSDM-N, resulting in swelling and lysis [8]. Triggered by certain inflammasomes, pyroptosis releases a large number of inflammatory factors, leading to immune disorders such as autoimmune disease [9]. Pyroptosis also plays a complex role in cancers: on the one hand, pyroptosis can inhibit the oncogenesis and progression of tumors; on the other hand, the inflammatory microenvironment formed by pyroptosis provides suitable conditions for tumor growth [10]. Previous studies have shown that pyroptosis is more associated with the cancers that develop as a result of inflammation, such as liver cancer [11], esophageal cancer [12], and colorectal cancer (CRC) [12]. Accounted for approximately 5% of CRC cases, CAC is developed from chronic UC [13]. Given the existing findings, pyroptosis plays a potentially important role in the inflammatory processes; however, the mechanism of pyroptosis in UC, the relationship between pyroptosis and CAC, and the clinical value of pyroptosis-related genes (PRGs) remain unknown.

The prognostic value of PRGs in cancers (such as breast cancer [14], lung cancer [15], and glioma [16]) has been extensively studied in recent years, whereas the value of PRGs remains unclear in UC. Biomarkers or gene signatures are of great importance in predicting disease, and more and more studies applied various methods to explore the significant biomarkers. For example, weighted gene coexpression network analysis (WGCNA) can search gene modules with coexpression and identify the correlations between the modules and phenotypes [17]. Logistic regression is often used to explore the risk factors that cause disease and predict the probability of disease occurrence based on the risk factors [18].

In this study, we comprehensively evaluated the expression profiles of PRGs and obtained the immune landscape between active and inactive UC. WGCNA and logistic regression analysis were applied to generate the pyroptosis-related signature (PR-signature) and PR-Score, which were significantly associated with active UC. We demonstrated that the PR-Score had a powerful diagnostic capability for active UC. Also, we reported the associations of the PR-signature with the anti-TNF therapy response and the occurrence of CAC. The levels of the PR-signature genes were validated in cell and animal models that mimic UC/CAC pathologies in vitro and in vivo. Our study will help clarify the role of pyroptosis in the progression and treatment of UC, thus optimizing personalized treatment.

## 2. Materials and Methods

### 2.1. Dataset Collection

**Figure 1:**
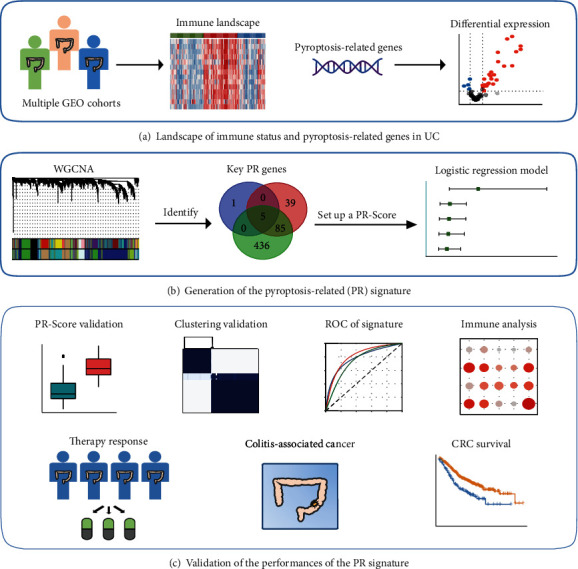
Research flow chart of this study. UC: ulcerative colitis; GEO: Gene Expression Omnibus; PR: pyroptosis-related; WGCNA: weighted gene coexpression network analysis; ROC: receiver operating characteristic; CRC: colorectal cancer.

The workflow of our study is shown in Figure 1. The Gene Expression Omnibus (GEO) database (http://www.ncbi.nlm.nih.gov/geo/) was used to download the microarray datasets. Detailed information of all datasets and included samples is shown in Table S1. Briefly, GSE13367, GSE38713, GSE48958, and GSE53306 were consisted of the training set, including 53 normal, 54 active UC, and 44 inactive UC colon mucosa samples. The “sva” R package was used to remove batch effects between different datasets. Principal component analysis (PCA) was used to detect the results by “prcomp” R function (Figure S1). Additionally, GSE75214 and GSE94648 were selected as validation sets. The GSE75214 contained 22 normal, 74 active UC, and 23 inactive UC mucosa samples, and the GSE94648 included 17 active UC and 8 inactive UC blood samples. Moreover, GSE111889 was obtained for clustering validation, including 73 UC mucosa samples.

To analyze the correlation between the PR-Score and therapy response, we searched several datasets with UC patients treated with anti-TNF agents, including GSE92415 (golimumab), GSE16879 (infliximab), and GSE23597 (infliximab).

Furthermore, to analyze the relationships of the PR-signature with CAC and CRC, two CAC datasets (GSE4183 and GSE47908) from human tissues and a mouse model of colitis-cancer (GSE31106) were collected. And two CRC cohorts with survival information of patients, GSE39582 and TCGA-COAD (The Cancer Genome Atlas—colon adenocarcinoma, https://portal.gdc.cancer.gov/), were also included.

### 2.2. Single-Sample Gene-Set Enrichment Analysis (ssGSEA)

The relative abundances of 28 immune cells in the colon mucosa tissue of normal control and active/inactive UC were calculated by ssGSEA. Besides, we selected some representative gene sets for inflammatory pathways from MSigDB (http://www.broad.mit.edu/gsea/msigdb/). The signatures of tumor immune-related pathways were collected from previous research [19]. The ssGSEA algorithm was also used to calculate the enrichment scores of these gene sets or signatures via the “GSVA” R package.

### 2.3. Identification of Differentially Expressed PRGs

Seventy-five PRGs were retrieved from previous publications [20, 21], and they were presented in Table S2. The “limma” R package was used to identify differentially expressed genes between active UC samples and inactive UC samples from PRGs. Those genes with the threshold adjusted *P* value (adj.*P*) < 0.05 and |log2 (fold change)| ≥ 1.0 were selected as differentially expressed PRGs.

### 2.4. Construction of WGCNA and Identification of PR-Signature

The training set was used to a coexpression network via the “WGCNA” R package. At first, Pearson's correlation matrices were calculated for all paired genes and a weighted adjacency matrix was constructed. A value of *β* = 5 scale (*R*^2^ = 0.88) was applied to build a scale-free network (Figure S2). Then, the adjacency matrix was converted to a topological overlap matrix (TOM) to prepare for hierarchical clustering analysis [22]. The gene modules were identified using a dynamic tree cut algorithm with a minimum size of 30, and gene modules were clustered according to the cutoff value of 0.32. The key module was identified by both the highest absolute value of module significance (MS), and the correlation between active UC and module eigengene (ME). Meanwhile, the hub genes in the key module were selected by a cutoff of the absolute value of Pearson's correlation (|cor.Standard|) > 0.55. By intersecting with the differentially expressed PRGs, the overlapping genes were identified as key PRGs and composed a pyroptosis-related signature (PR-signature).

### 2.5. Generation of Logistic Regression Model and Pyroptosis-Related Score (PR-Score)

Univariate logistic regression analysis was used to validate the diagnostic abilities of the PRGs in the PR-signature for active UC. Based on the PR-signature genes, multivariate logistic regression model was constructed to establish a PR-Score using the training set.

The PR-Score was calculated as follows:

(1) $$\begin{array}{c} \text{PR}-\text{Score}=\Sigma \;\left(\text{Expi}*\text{Coefi}\right)+b, \end{array}$$

where Expi, Coefi, and *b* denote expression of each gene, the risk coefficient, and the constant term in the logistic regression equation, respectively.

Based on the median of PR-Score, patients with UC could be separated into high- and low-PR-Score groups for further analysis.

### 2.6. Consensus Clustering Analysis of UC Samples

Based on the expressions of the PR-signature genes, we used consensus unsupervised clustering to classify UC patients into clusters to investigate whether the PR-signature can distinguish active UC from inactive UC. The “ConsensusClusterPlus” package was used to perform the above steps [23]. The key operating parameters included 80% item resampling, a maximum evaluated *k* of 9, and 1000 repetitions for guaranteeing the stability of classification.

### 2.7. Cell Line and Treatment

Normal colon mucosal epithelial cell line NCM460 was purchased from the China Center for Type Culture Collection (Wuhan, China), and cells had been authenticated for STR profiling and tested for mycoplasma by the vendor. All the cells were cultured in RPMI 1640 medium (HyClone, USA) containing 10% fetal bovine serum (FBS, HyClone, USA), 100 U/ml penicillin, and 100 mg/ml streptomycin (Genom, China), at 37°C with 5% CO_2_. 50 ng/ml TNF-*α* (PeproTech, USA) treated cells for 24 h to construct a cell model that mimics the active UC pathology in vitro.

### 2.8. Quantitative Reverse Transcription Polymerase Chain Reaction (qRT-PCR) Assays

Total RNA was extracted from NCM460 using TRIzol reagent (Invitrogen, Carlsbad, CA, USA). RNA quantity was determined by NanoDrop 2000c (Thermo Scientific, Waltham, MA, USA). For qRT-PCR, 1 *μ*g RNA was reverse transcribed to cDNA using a Reverse Transcription Kit (Toyobo, Osaka, Japan). The qRT-PCR assays were conducted on LightCycler® 96. Target gene expression was normalized against GAPDH. The expression levels of mRNA were calculated using the comparative CT (2-*ΔΔ*CT), and all experiments were performed with three biological replicates. The primer sequences are listed in Table S3.

### 2.9. The Models of DSS-Induced Colitis and AOM/DSS CAC

Nine male C57BL/6 mice, aged 8 weeks, were purchased from the Beijing Vital River Laboratory Animal Technology Company (Beijing, China). All mice were housed in specific pathogen-free conditions at the Animal Experimental Center of Zhongnan Hospital of Wuhan University. Mice were grouped into DSS- (dextran sulfate sodium-) induced colitis model and AOM (azoxymethane)/DSS-induced CAC model. The detailed processes of establishing the models were carried out as previously described [24–26]. Briefly, 3% DSS was added to the drinking water to induce colitis for 7 consecutive days. On day 8, the mice were sacrificed, and colon samples were collected. To establish an AOM/DSS model, 8-week-old male mice were administered with a single intraperitoneal injection (i.p.) of 10 mg/kg AOM (Sigma-Aldrich, MO, USA), followed by seven days of regular diet and water ad libitum. Mice were then administered with three cycles of 1.5% DSS (Sigma-Aldrich, MO, USA) for seven days and drinking water for 14 days. After three cycles, they were sacrificed, and colon samples were collected.

All animals received humane care, and the study protocol was approved by the Animal Ethics Committee of Wuhan University (Animal Use Protocol Number: 2019157).

### 2.10. Mouse Tissue Processing and Immunohistochemistry (IHC)

Colon tissues were fixed in 10% neutral-buffered formalin (Sigma-Aldrich, USA) and embedded in paraffin. Tissue sections were sliced from paraffin blocks into 4 *μ*m thick slices. The slices were further used for IHC test.

IHC was used to measure the levels of CASP5, GZMB, GBP1, IL1B, and IRF1 in colonic tissues as previously described [27]. The primary antibodies applied were CASP 5 (1 : 400, Boster, A05259-4), GZMB (1 : 3000, abcam, ab255598), GBP1 (1 : 300; Proteintech, 15303-1-AP), IL1B (1 : 500; abcam, ab283818), and IRF-1 (1 : 400, Cell Signaling Technology, 8478). CASP 5, IL1B, and IRF1 were performed heat-mediated antigen retrieval with Tris/EDTA buffer pH 9.0; others were with citrate buffer pH 6.0. We semiquantified the expression levels of the PR-signature genes using an immune-reactive score (IRS) method [28]. Briefly, the proportion of positively stained cells was graded as 0 (0%), 1 (0-5%), 2 (6-10%), 3 (11-50%), or 4 (51-100%), and the staining intensity was expressed as 0 (none), 1 (weak), 2 (intermediate), or 3 (strong). Both values were multiplied to obtain an IRS between 0 and 12. The evaluation was carried out independently by two observers.

### 2.11. Statistical Analyses

Receiver operating characteristic (ROC) curve was used to show the diagnostic ability of PR-Score and the clustering ability of PR-signature for distinguishing active UC from inactive UC. The “pROC” R package was used to plot the ROC curve. The “survival” R package was used to investigate the correlation of PR-Score and CRC survival outcomes, and the optimal cutoff point of PR-Score was determined using the “survminer” R package. The correlation between PR-Score and clinicopathologic features was analyzed using the *χ*^2^ test. Unpaired two-tailed Student's *t*-test or one-way ANOVA was used for more than two comparison groups. The results are expressed as the mean ± SEM at least three replicates. IBM SPSS version 25.0 was used to conduct logistic regression analyses, and other statistical analyses were performed using R version 4.1.0 and GraphPad Prism 9. All results were considered to be statistically significant at *P* < 0.05.

## 3. Results

### 3.1. Landscape of Immune Status and Differential Expression of PRGs in UC

**Figure 2:**
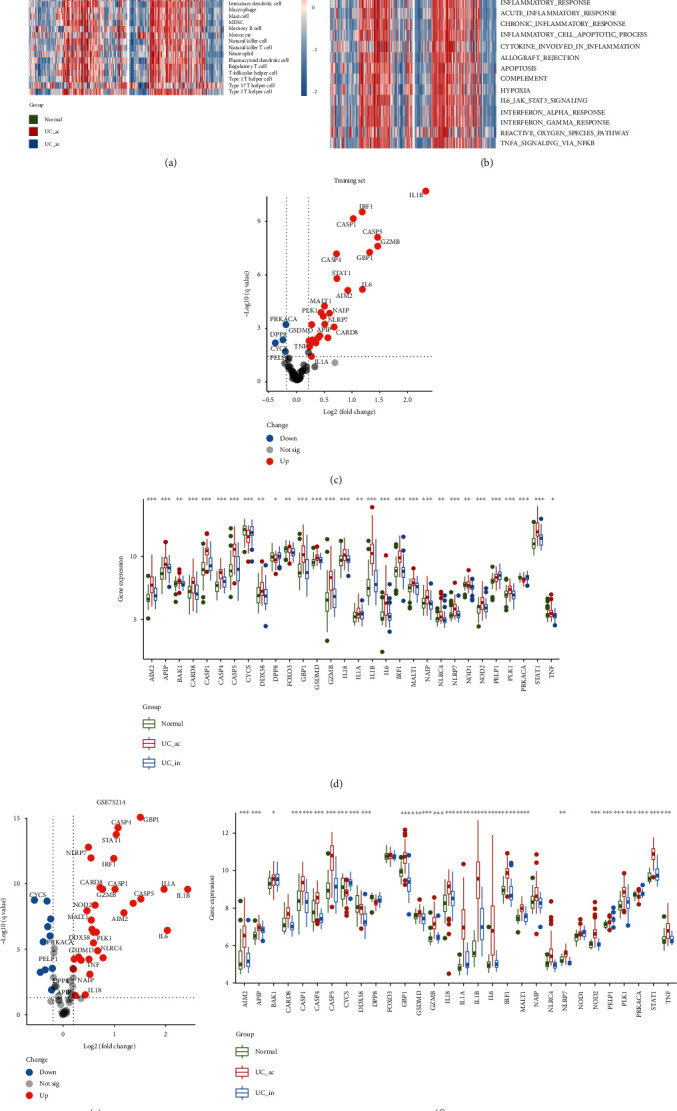
Landscape of immune status and pyroptosis-related genes (PRGs) in UC. (a) The distributions of 28 immune cells in normal and active/inactive UC samples. Red represents abundant infiltration and blue represents immune cell desert. (b) GSVA showed the distributions of the inflammation-related pathways in normal and active/inactive UC samples. Red represents activation of biological pathways and blue represents inhibition of biological pathways. (c) The volcano plot displayed the expression changing information of 75 PRGs between active UC and inactive UC samples in the training set. (d) Expression distributions of the 30 altered PRGs in normal and active/inactive UC samples in the training set. (e) The volcano plot showed the expression changing information of 75 PRGs between active UC and inactive UC samples in GSE75214. The marked genes are the 30 altered PRGs in the training set. (f) Expression distributions of the 30 altered PRGs in normal and active/inactive UC samples in GSE75214. UC: ulcerative colitis; ac: active; in: inactive. ^∗^*P* < .05, ^∗∗^*P* < .01, ^∗∗∗^*P* < .001, and ^∗∗∗∗^*P* < .0001. ns: not significant.

We first explored the differences of the immune microenvironment of UC and normal samples. As shown in the heat map (Figure 2(a)), both in the training set and GSE75214, active UC samples had more abundant immune cell infiltration than normal control, while inactive UC was more similar to the normal, showing a status of immune desert. Meanwhile, the enrichment scores of the inflammatory-related pathways showed similar distributions in normal and active/inactive samples as the immune cell infiltration (Figure 2(b)). Active UC was not only active in the pathways of inflammatory response as well known, but also in IL6-JAK-STAT3 signaling, interferon-*α*/*γ* response, and TNF-*α* signaling via NF-*κ*B. In contrast, these pathways were silent in the inactive UC, similar to the status of normal tissues.

Next, to investigate the relationship of pyroptosis and the occurrence of active UC, there were 75 PRGs involved in the study. Differentially expressed analysis (active UC vs. inactive UC) identified 30 PRGs with altered expression in the training set, where only a few genes were downregulated while a large proportion of them was upregulated (Figure 2(c) and Table S4), indicating upregulation of the pyroptosis regulators was more related to active UC. The levels of the altered PRGs in normal and inactive UC were similar, but significantly different from the active UC (Figure 2(d)). In GSE75214, these altered PRGs also showed consistent regulatory trends (Figures 2(e) and 2(f) and Table S5). Together, we found that the trends of PRG alteration were similar to the trends of immune status in UC, suggesting pyroptosis was correlated with the progression of UC.

### 3.2. Generation of PR-Signature and PR-Score

**Figure 3:**
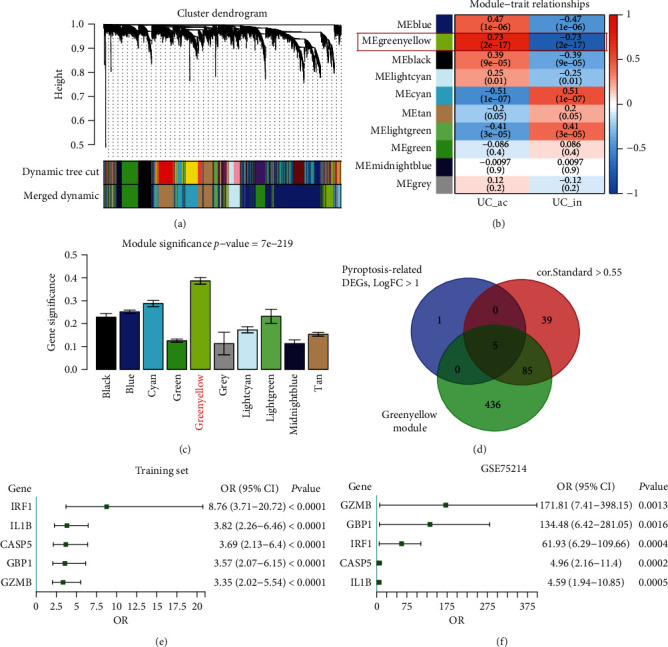
Identify the key PRGs and establish the PR-Score. (a) Module clustering dendrogram based on a dissimilarity measure (1-TOM). (b) Heat map of the correlations between MEs and active/inactive UC. (c) Distributions of average gene significance and errors in the modules related to active ulcerative colitis. (d) Venn diagram showed the overlapping genes between differentially expressed PRGs, the genes of greenyellow module, and the genes with ∣cor.Standard | >o.55 in WGCNA. Logistic regression analysis for the key PRGs in the training set (e) and GSE75214 (f). ME: module eigengene; UC: ulcerative colitis; ac: active; in: inactive; DEGs: differentially expressed genes; OR: odds ratio.

A WGCNA was then constructed to explore the crucial genes related to active UC. We identified ten modules in the module classification through WGCNA (Figure 3(a)). The ME showed the greenyellow module was most positively relevant to the active UC (*r* = 0.73, *P* = 2*e* − 17) (Figure 3(b)). In addition, the MS of the greenyellow module was higher than other modules (Figure 3(c)). Thus, our data indicated that the greenyellow module was the most associated with the occurrence of active UC. Meanwhile, under the absolute value of Pearson's correlation coefficients (|cor. Standard|) higher than 0.55 (Table S6) and |log2 (fold change)| ≥ 1.0 of differential expression analysis for PRGs as mentioned above, a Venn diagram identified CASP5 (caspase 5), GBP1 (guanylate binding protein 1), GZMB (granzyme B), IL1B (interleukin 1 beta), and IRF1 (interferon regulatory factor 1) (Figure 3(d)) as key PRGs to compose a PR-signature.

Then, to confirm the PR-signature has a remarkable positive correlation with active UC, univariate logistic regression analysis was conducted for the PR-signature genes both in the training set and GSE75214 (Figures 3(e) and 3(f) and Table S7). Results showed that all 5 PR-signature genes could predict active UC well. Based on the PR-signature genes, multivariate logistic regression model was constructed to develop a PR-Score to distinguish active UC and inactive UC samples using the training set. The PR − Score = CASP5^∗^ (0.562) + IL1B^∗^ (0.956) + GZMB^∗^ (0.419) + IRF1^∗^ (0.885) + GBP1^∗^ (0.650) − 19.461.

### 3.3. Validation of the Performances of the PR-Score in UC

**Figure 4:**
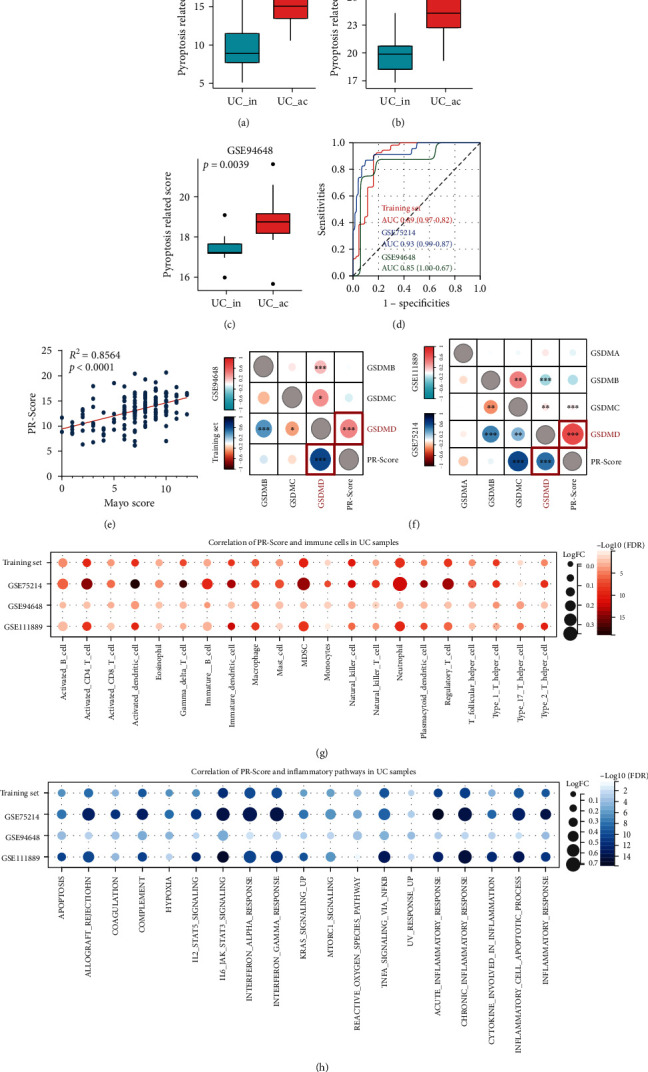
Correlation between PR-Score, expression of GSDM family members, and immune status. Differences of the PR-Score in active UC and inactive UC samples in the training set (a), GSE75214 (b), and GSE94648 (c). (d) The ROC curve showed the diagnostic efficiency of the PR-Score in three datasets. (e) The correlation of the PR-Score and Mayo score in GSE92415. (f) PR-Score was most positively associated with the expression of GSDMD in all datasets (left: the training set and GSE94648; right: GSE75214 and GSE111889). (g) Correlations of PR-Score and immune cells in UC samples in four datasets. (h) Correlations of PR-Score and inflammation-related pathways in UC samples in four datasets. UC: ulcerative colitis; ac: active; in: inactive; AUC: area under the curve. ^∗^*P* < .05, ^∗∗^*P* < .01, ^∗∗∗^*P* < .001, and ^∗∗∗∗^*P* < .0001. ns: not significant.

In order to validate the performance of PR-Score in UC, several datasets were used for analysis. Both in the training set and GSE75214, PR-Scores were significantly higher in the group of active UC than the inactive UC (Figures 4(a) and 4(b)). Interestingly, in GSE94648, a set with blood samples, PR-Score was also significantly high in the active UC, indicating PR-Score in blood also can distinguish the samples with active UC from the inactive UC (Figure 4(c)). The ROC curve showed that PR-Score had a great power in diagnosing active UC in three datasets, all with AUC > 0.8 (Figure 4(d)). In addition, PR-Score had a strong positive correlation with the Mayo score in GSE92415 (Figure 4(e)) and GSE94648 (Figure S3(a)). The Mayo score is an indicator of the severity of UC based on the endoscopic presentation, and higher score indicates severer disease [29]. As for other clinicopathologic features, PR-Score was associated with the lesion location of UC (Table S8-9).

Since the GSDM family genes play important roles in pyroptosis [8], we next analyzed the correlations of PR-Score and the GSDM family members. As shown in Figure 4(f), we observed that PR-Score was most positively associated with the expression of GSDMD, which was identified as the most important pyroptosis executioner among the GSDM genes [30].

In terms of affecting immune status, we found that PR-Score was significantly positively related to immune cell infiltration, including neutrophils, myeloid-derived suppressor cells, activated CD4 T cells, and regulatory T cells (Figure 4(g)), which indicated that PR-signature genes could be involved in the inflammation of UC by regulating pyroptosis of these cells. Meanwhile, PR-Score had positive correlations with multiple inflammatory pathways (Figure 4(h)), among which the IL6-JAK-STAT3 signaling, interferon-*α*/*γ*, and chronic inflammatory response were most significant.

### 3.4. PR-Signature Was an Effective Classifier for Active/Inactive UC and Inflamed/Uninflamed Immune Status

**Figure 5:**
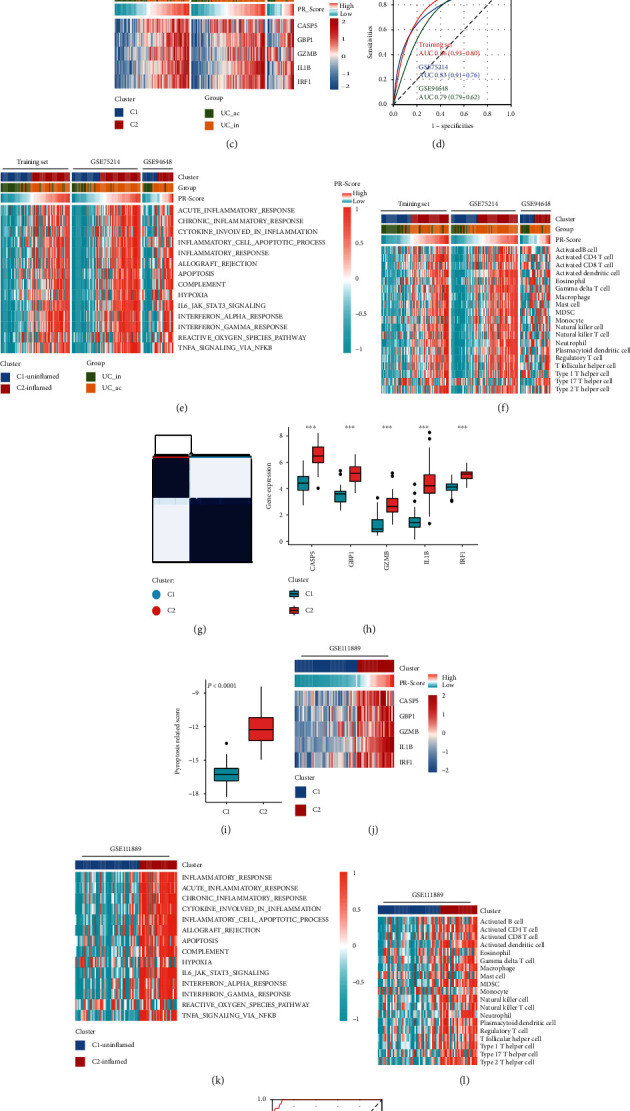
The capacity of PR-signature in classifying active/inactive UC and inflamed/uninflamed immune status. (a) Based on the PR-signature, UC samples of the training set were reclassified into two clusters using unsupervised consensus clustering. (b) Distributions of two clusters in active UC and inactive UC. (c) Distributions of active/inactive UC samples, PR-Score, and expression of the five key PRGs in two clusters. (d) The ROC curve showed the diagnostic efficiency of the PR-signature in three datasets. (e) Differences of activation of inflammatory-related pathways in two clusters. (f) Differences of immune cell infiltration in two clusters. (g) UC samples of GSE111889 were classified into two clusters based on the PR-signature using unsupervised consensus clustering. (h) Expression differences of the five key PRGs in two clusters. (i) Differences of the PR-Score in two clusters. (j) Distributions of PR-Score and expression of the five key PRGs in two clusters. (k) GSVA showed the distributions of the inflammatory-related pathways in two clusters. Orange represents activation of biological pathways and blue represents inhibition of biological pathways. (l) The distributions of immune cells in two clusters. Orange represents abundant infiltration and blue represents immune cell desert. (m) The ROC curve showed the diagnostic efficiency of the PR-signature for two clusters. UC: ulcerative colitis; ac: active; in: inactive; AUC: area under the curve.

By means of unsupervised clustering, we validated the classification ability of PR-signature for active and inactive UC patients. Based on the PR-signature genes, UC patients were reclassified into two clusters (Figure 5(a) and Figure S3 (b, d)). Notably, we found that the major proportion of cluster 2 (C2) was active UC, while the major proportion of cluster 1 (C1) was inactive UC (Figure 5(b) and Figure S3(e)). As the heat map shows (Figure 5(c)), active UC, higher PR-Score, and higher expressions of the PR-signature genes were more likely to concentrate in C2 than C1, so we suggested C2 might represent the samples with active UC in the clustering. The ROC curve revealed that the PR-signature also had good diagnostic capability for active UC (Figure 5(d)), but it was slightly inferior to PR-Score (Figure 4(d)). As shown in Figures 5(e) and 5(f), the samples in C2 had more abundant infiltration of immune cells and higher enrichment scores of the inflammation-related pathways than C1 across three datasets, so we defined C2 as the inflamed class while C1 as the uninflamed class in UC. Also, this finding further confirmed the differences of immune status between active and inactive UC.

Likewise, the patients in GSE111889 were not distinguished between active and inactive UC, but we found that the UC samples could also be well classified into two clusters based on the PR-signature (Figure 5(g) and Figure S3(f)). The UC samples in C2 had higher expressions of the PR-signature genes and higher PR-Scores than C1 (Figures 5(h) and 5(j)). Based on the knowledge from the results above, this C2 was closer to active UC. Besides, compared with C1, C2 had more abundant infiltration of immune cells and higher enrichment scores of inflammation-related pathways at the same time (Figures 5(k) and 5(l)). Therefore, C2 was reasonably identified as the inflamed class while C1 was the uninflamed class. Collectively, the clustering of C2 and C1 was likely equivalent to the classification of active and inactive UC, respectively. And the ROC curve showed that PR-Score could distinguish C1 and C2 well (Figure 5(m)). Collectively, the PR-signature could serve as an effective classifier for active/inactive UC and inflamed/uninflamed status.

### 3.5. Lower PR-Score Was Associated with a Better Response to Anti-TNF Therapy for Patients with UC

**Figure 6:**
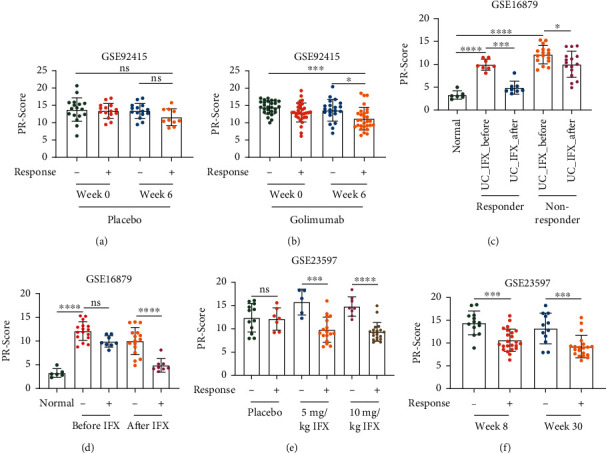
Differences of the PR-Score in responders/nonresponders with anti-TNF treatment. Based on the GSE92415 dataset, UC patients were treated with placebo (a) and golimumab (b) for 6 weeks, respectively. The PR-Score was compared. According to the GSE16879 dataset, the PR-Score was compared between UC patients before and after IFX treatment (c), or between responders and nonresponders (d). Based on the GSE23597 dataset, compare the effects of IFX doses (e) and the time of IFX administration (f) on the PR-Score in patients with IFX response. UC: ulcerative colitis; IFX: infliximab. ^∗^*P* < .05, ^∗∗^*P* < .01, ^∗∗∗^*P* < .001, and ^∗∗∗∗^*P* < .0001. ns: not significant.

Anti-TNF agents are widely used to treat UC, but the effects vary from person to person [31]. We next investigated the relationship of PR-Score with the anti-TNF therapeutic response. Based on the GSE92415 dataset, PR-Score did not differ between placebo groups (Figure 6(a)) but was lower in the responders than in the nonresponders after 6 weeks of golimumab treatment (Figure 6(b)). Similarly, in GSE16879, both before and after infliximab (IFX) treatment, PR-Score was lower in the responders but higher in the nonresponders (Figure 6(c)). And interestingly, PR-Score was decreased after IFX treatment compared with before treatment, which was more significant in the responders (Figure 6(d)). Importantly, in GSE23597, PR-Score was significantly lower in the responders than the nonresponders, despite of dose or time administration (Figures 6(e) and 6(f)). In summary, the responders in anti-TNF treatment had lower PR-Score and PR-Score showed a decline after anti-TNF treatment. These findings suggested that the PR-signature genes were connected to the anti-TNF therapy and PR-Score could predict the anti-TNF therapeutic responses.

### 3.6. High PR-Score Suppressed the Occurrence of CAC and Improved the Survival Outcomes for Patients with CRC

**Figure 7:**
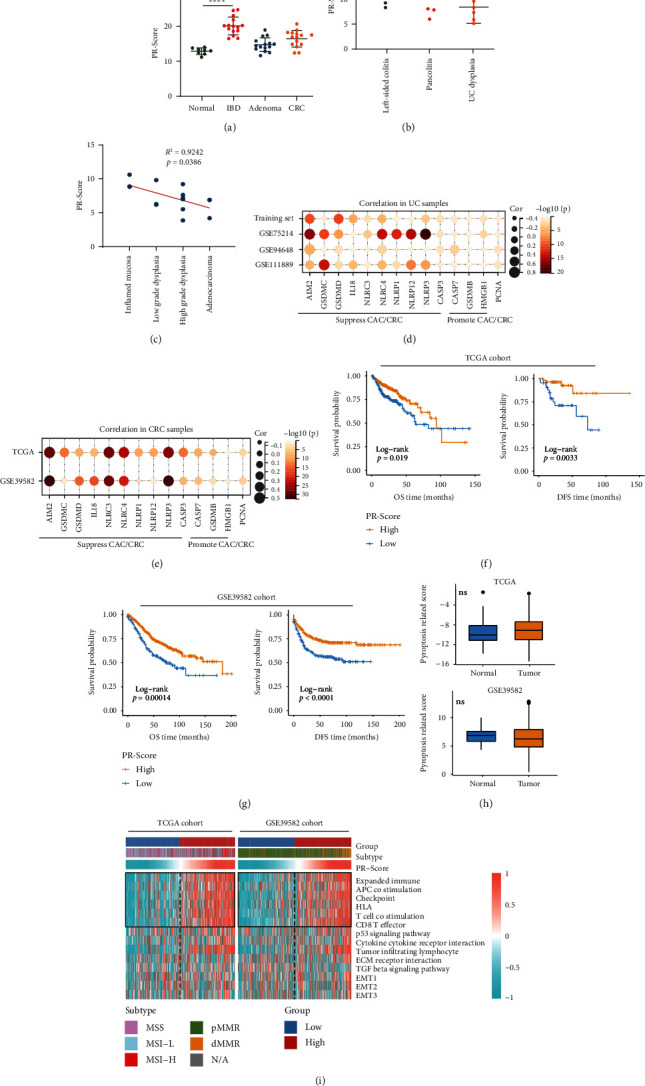
Relationships between the PR-Score/PR-signature, colitis-associated colorectal cancer (CAC), and colorectal cancer (CRC). (a) Comparison of the PR-Score in normal, IBD, adenoma, and CRC tissues using GSE4183. (b) Comparison of the PR-Score in colitis and UC-dysplasia tissues using GSE47908. (c) Correlation of the PR-Score and the progression from inflamed dysplasia to adenocarcinoma. Correlation of the PR-Score and the molecules that suppress/promote tumors in UC samples (d) and CRC samples (e). The effect of PR-Score on the OS and DFS of CRC in TCGA (f) and GSE39582 (g). (h) Differences of PR-Score between normal and tumor in TCGA and GSE39582. (i) Correlations of the PR-Score and the tumor immune-related pathways and CRC subtypes in TCGA and GSE39582. IBD: inflammatory bowel disease; CAC: colitis-associated colorectal cancer; CRC: colorectal cancer; UC: ulcerative colitis; OS: overall survival; DFS: disease-free survival; MSS: microsatellite stability; MSI-H: high microsatellite instability; MSI-L: low microsatellite instability; pMMR: proficient-mismatch-repair; dMMR: deficient-mismatch-repair; N/A: not applicable. ^∗^*P* < .05, ^∗∗^*P* < .01, ^∗∗∗^*P* < .001, and ^∗∗∗∗^*P* < .0001. ns: not significant.

We next analyzed the associations of PR-Score with occurrence of CAC and tumor immunity of CRC. Notably, PR-Score was lower in adenoma and CRC compared with IBD tissues in GSE4183 (Figure 7(a)). Consistently, in GSE47908, PR-Score was also lower in UC-dysplasia than colitis samples (Figure 7(b)). In addition to these findings, in GSE31106, a mouse model, PR-Score was negatively correlated with the progressions from inflamed dysplasia to adenocarcinoma (Figure 7(c)). In brief, PR-Score was decreased in the samples with UC-related CRC, inferring the PR-signature genes might protect body from UC-associated cancer occurring. Meanwhile, PR-Score was positively correlated with the expressions of the molecules that suppress CAC/CRC but had no significant correlations with the tumor-promoting molecules (Figures 7(d) and 7(e)). AIM2 was the most significant among the molecules both in UC and CRC samples. These data provided further evidences that PR-signature could inhibit tumorigenesis. In the patients with CRC, PR-Score was associated with the clinicopathologic features such as tumor stage, lymphatic invasion, and KRAS mutation (Table S10-11). Additionally, it is worth noting that higher PR-Score inferred better overall survival (OS) and disease-free survival (DFS) outcomes, which were consistent in the TCGA and GSE39582 cohorts (Figures 7(f) and 7(g)), also indicating that the PR-signature genes tended to play roles in suppressing CRC. The PR-Score had no significant difference between normal and tumor tissues (Figure 7(h)). By analyzing tumor immune-related pathways, we found that the pathways including “expanded immune,” “APC costimulation,” and “T cell costimulation” were significantly active in the high-PR-Score group; and the immune molecules including HLA, checkpoint, and CD8 T cell effector were also more active in the high-PR-Score group (Figure 7(i)). Together, the PR-signature played a potential antitumor role in CRC progression. The heat map also displayed the distribution of the subtypes of CRC between the high- and low-PR-Score groups (Figure 7(i)). The MSI-H/dMMR (high microsatellite instability/deficient-mismatch-repair) CRC was more likely to concentrate in the high-PR-Score group, while the MSS/pMMR (microsatellite stability/proficient-mismatch-repair) was more likely to concentrate in the low-PR-Score group, indicating the PR-signature was related to the microsatellite instability.

### 3.7. Validation of the Levels of the PR-Signature Genes In Vitro and In Vivo

**Figure 8:**
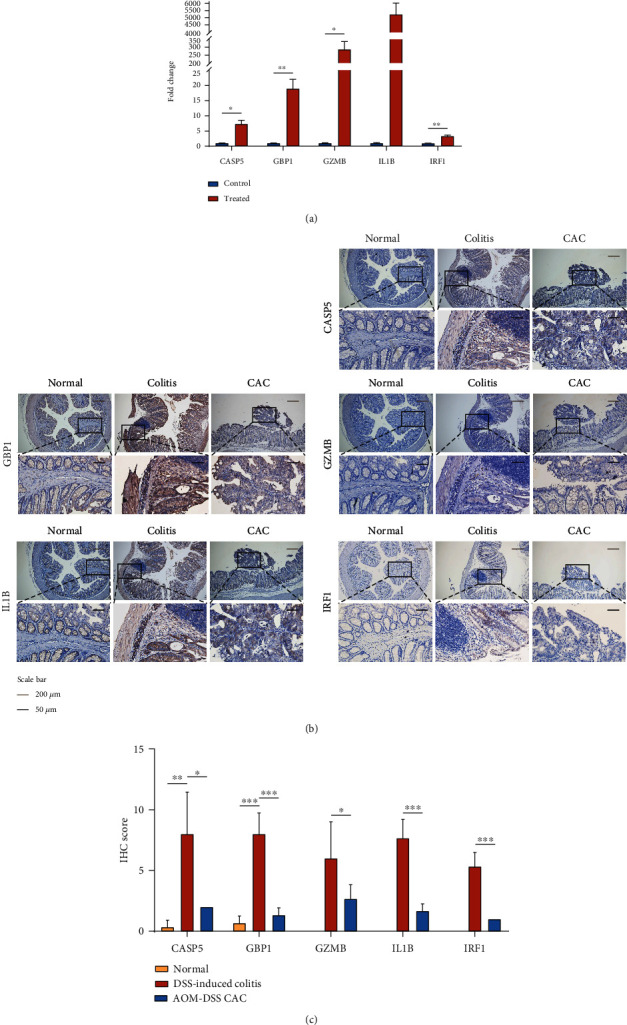
Validation of the levels of the PR-signature genes in vitro and in vivo. (a) The expressions of 5 PR-signature genes in normal NCM460 cells and TNF-*α*-stimulated inflammation cells. (b) Representative IHC images showing the expressions of 5 PR-signature genes in normal (left), DSS-induced colitis (middle), and AOM/DSS-induced CAC (right) tissues of mice. (c) Semiquantification of IHC staining of 5 PR-signature genes in normal, DSS-induced colitis, and AOM/DSS-induced CAC tissues of mice. CAC: colitis-associated colorectal cancer; IHC: immunohistochemistry. ^∗^*P* < .05, ^∗∗^*P* < .01, and ^∗∗∗^*P* < .001.

Since the above conclusions were based on bioinformatics analysis, further validation was required to provide sufficient proof that the PR-signature was positively associated with the occurrence of the active UC but negatively correlated with the development of CAC. Thus, we validated these results in vitro and in vivo experiments. As shown in Figure 8(a), the expressions of CASP5, GBP1, GZMB, IL1B, and IRF1 were significantly upregulated in the TNF-*α*-stimulated NCM460 cells. Meanwhile, we also compared the associations of the PR-signature genes and the occurrence of UC and CAC in mouse models. The results showed that the protein levels of CASP5, GBP1, GZMB, IL1B, and IRF1 were significantly increased in the active UC tissues compared with normal controls while markedly reduced in CAC tissues compared to UC (Figures 8(b) and 8(c) and Figure S4). These data confirmed that the high level of PR-signature was associated with the occurrence of UC and suppression of CAC.

## 4. Discussion

Active UC and inactive UC are two different clinical stages of disease, and our study revealed the distinctions in the immune microenvironment between them. As a proinflammatory cell death, pyroptosis was potentially involved in the progression of inactive UC to the active stage. In this study, we generated a PR-Score/PR-signature that was associated with the occurrence of active UC and could distinguish active UC from inactive UC well. The PR-Score/PR-signature consisting of CASP5, GBP1, GZMB, IL1B, and IRF1 was closely related to the immune status, anti-TNF therapy response, and CAC suppression. Our findings may provide new markers for predicting the development of active UC and CAC and new targets for individualized treatment of UC patients.

One of the PR-signature genes identified by our study, IL1B, is a proinflammatory cytokine that plays an important role in many physiological and pathological processes [32, 33]. Pro-IL1B and GSDMD can be proteolytic processed by activated CASP1, which leads to the formation of the GSDMD pore, the release of mature IL1B, and eventually pyroptosis [34]. Li et al. clarified that activation of NF-*κ*B signaling can exacerbate colonic inflammation in UC by releasing IL1B and inducing intestinal epithelial cell pyroptosis [35]. Therefore, the role of IL1B in active UC is relatively clear, and our study provides consistent and further evidences. Like CASP1, CASP5 is a member of the caspase family. CASP1 is an important signaling molecule in promoting inflammation in UC [34], but the role of CASP5 in UC is less reported. It is worth mentioning that CASP5 was highly expressed in the stromal compartment of inflammatory UC and CRC tissues [36]. Also, CASP5 is essential for heme-induced IL1B release and the heme-induced activation of CASP5 is a key mediator of inflammation in macrophages [37]. Our study points out that CASP5 was a key pyroptosis regulator associated with the occurrence of active UC, providing more insights for further studies. IRF1 is required for IFNs to induce MHC class I proteins and prime CD8+ T cell responses [38]. IRF1 regulates IL-15 expression and controls the development of NK1+ T cells, NK cells, and CD8+ intestinal intraepithelial lymphocytes [39]. Thus, IRF1 is close to immune activation and inflammatory response. Fang et al. reported that IRF1 was upregulated in pediatric IBD and experimental colitis [40]. During intestinal inflammation, the activation of TNF-*α* upregulates the expression of IRF1, resulting in aggravating apoptosis of the intestinal epithelial cells [41]. Our study also identified IRF1 as a key pyroptosis molecule involved in the inflammatory activity of UC. GZMB is a member of granzyme (GZM) family and also possesses the cytotoxic activities [42]. GZMB and CASP1 together activate GSDME to cause cell pyroptosis and participate in the inflammatory activity [43]. Significant upregulated expression of GBP1 was found in inflammatory disease such as rheumatoid arthritis [44]. Additionally, Zhang et al. observed that inflammatory macrophage was significantly enriched in severe COVID-19 samples and showed significantly elevated levels of proinflammatory genes, including GBP1 and IL1B [45]. Here, we demonstrated that the five PR-signature genes were highly upregulated in the inflammation cell model and DSS-induced colitis samples. Therefore, the five PR-signature genes were all proven to be positive pyroptosis regulators associated with the occurrence of active UC. Meanwhile, PR-Score was positively correlated with the interferon-*α*/*γ*, chronic inflammatory response, and IL6/JAK/STAT3 pathways, further proving that the PR-signature genes helped with inflammation.

This PR-signature contains molecules that are clearly associated with UC inflammation (IL1B, CASP5, and IRF1) as well as novel molecules rarely reported in UC (GZMB and GBP1), which together may better predict and differentiate the active UC from inactive UC. More importantly, we validated the diagnostic power of the PR-signature/PR-Score in multiple datasets, including not only tissue sample sets but also a blood sample set (GSE94648). The PR-signature/PR-Score showed outstanding diagnostic ability and may be utilized to be a novel signature detecting the occurrence of active UC even in blood samples. We also revealed that the PR-Score had a significant positive correlation with GSDMD, which is the most important pyroptosis executioner [8]. Collectively, the PR-signature genes could participate in the development of active UC by mediating pyroptosis, but the specific mechanism still needs further exploration. Many studies have revealed that the pathogenesis of IBD involves various immune cells, such as neutrophil [46], innate lymphoid cells [47], and subsets of CD4+ T cells [48]. Notably, PR-Score was positively correlated with the infiltration of neutrophil, active CD4+ T cells, and regulatory T cell (one subset of CD4+ T cells) in our study, suggesting the PR-signature genes were involved in inflammatory activity of UC by enriching these immune cells. This correlation was weak in the blood samples (GSE94648) on account of the low levels of immune cells in blood.

Another important finding of our study is that PR-Score was significantly associated with the response to anti-TNF treatment. Tang et al. demonstrated that TNF-*α* blockade can induce macrophage pyroptosis by inhibiting the IRF1 pathway and thus alleviate the inflammatory activity [41]. Anti-TNF-*α* or an NF-*κ*B signaling pathway inhibitor would reduce the release of cytokines [49]. This can partially explain the decline of PR-Score after IFX and golimumab treatments. The responders of anti-TNF therapy had significantly lower PR-Score than the nonresponders, inferring low PR-Score could enhance and predict the response to anti-TNF antibodies. Consistently, Salvador-Martín et al. revealed [50] that GBP1 was downexpressed in responders compared with nonresponders in UC, and Lykowska-Szuber et al. reported [51] that IL1B was significantly downregulated in responders in Crohn's disease. Therefore, the PR-signature genes were potentially involved in the response process of anti-TNF therapy and can serve as promising predictors for anti-TNF therapy response in UC.

Pyroptosis is usually induced by inflammasome that is an important innate immune pathway [52, 53]. Recent studies demonstrated that inflammasome promote tumor progression in skin and breast cancer [54, 55]. However, on the intestinal mucosal surface, the intestinal barrier function and tumor surveillance depend on activation of inflammasome to product active IL1B and IL18 [52]. Previous studies showed that the inflammasome components provide protection against the development of colon cancer in the CAC model [56, 57]. In our study, PR-Score was negatively associated with the progress of inflamed dysplasia to adenocarcinoma, indicating that the PR-signature played a protective role in CAC. The in vivo experiments in CAC mouse model proved that the levels of PR-signature genes were indeed reduced in the CAC samples compared to UC. Besides, we found that PR-Score was positively correlated with the CAC/CRC inhibitory molecules, among which AIM2 was the most significant. Researches demonstrated that the expression of Aim2 is markedly reduced in tumor-associated tissues of mice, and mice lacking Aim2 are hypersusceptible to both colitis-associated and spontaneous colorectal tumorigenesis [58, 59]. This indirectly confirmed the effect of the PR-signature on suppressing CAC. Our study also found that the high-PR-Score group showed more MSI-H CRC and more active antitumor-related pathways/molecules. Collectively, high-PR-Score may be a protective factor against CRC and thus improve the survival outcomes.

## 5. Conclusions

In brief, we identified CASP5, GBP1, GZMB, IL1B, and IRF1 as key PRGs and established a novel PR-signature/PR-Score, which had great power to distinguish active UC from inactive UC. The in vitro and in vivo experiments validated that the PR-signature genes were overexpressed in UC pathologies. We also determined the therapeutic liability of the PR-signature genes in anti-TNF treatment for UC patients. The correlations between the PR-signature genes and CAC were clarified using public datasets and the CAC mouse model. Our study provided promising pyroptosis-related biomarkers for predicting the progressions of UC and CAC and guided individualized anti-TNF therapy for UC patients.

## Data Availability

Publicly available datasets were analyzed in this study. These data can be found here: https://portal.gdc.cancer.gov/ and https://www.ncbi.nlm.nih.gov/geo/. All processed data and R codes used in this study can be obtained from the corresponding authors on reasonable request.

## Supplementary Materials

Supplementary Materials Figure S1: the principal component analysis of before and after batch correction of all samples in the training set. Figure S2: determination of soft-thresholding power in the coexpression network. Figure S3: the correlation of the PR-Score and PR-clusters. Figure S4: representative IHC images showing the expressions of 5 PR-signature genes in normal, DSS-induced colitis, and AOM/DSS-induced CAC tissues of mice. Table S1: detailed information of all datasets and included samples. Table S2: summary of 75 recognized pyroptosis-related genes. Table S3: the primer sequences of CASP5, GBP1, GZMB, IL1B, and IRF1. Table S4: differential expression analysis of pyroptosis-related genes in the training set (active UC vs. inactive UC). Table S5: differential expression analysis of pyroptosis-related genes in GSE75214 (active UC vs. inactive UC). Table S6: standard weight of each gene in WGCNA. Table S7: logistic regression analysis for the key PRGs. Table S8: correlation of clinicopathologic characteristics and PR-Score in GSE111889. Table S9: correlation of clinicopathologic characteristics and PR-Score in GSE94648. Table S10: correlation of clinicopathologic characteristics and PR-Score in TCGA. Table S11: correlation of clinicopathologic characteristics and PR-Score in GSE39582.
