# Supplementary material for: Pyroptosis-Related Signature Predicts the Progression of Ulcerative Colitis and Colitis-Associated Colorectal Cancer as well as the Anti-TNF Therapeutic Response
Source: J Immunol Res. 2023 Jan 27;2023:7040113. doi: 10.1155/2023/7040113 (PMC9897931; doi:10.1155/2023/7040113)
Supplement: Supplementary Materials — Figure S1: the principal component analysis of before and after batch correction of all samples in the training set. Figure S2: determination of soft-thresholding power in the coexpression network. Figure S3: the correlation of the PR-Score and PR-clusters. Figure S4: representative IHC images showing the expressions of 5 PR-signature genes in normal, DSS-induced colitis, and AOM/DSS-induced CAC tissues of mice. Table S1: detailed information of all datasets and included samples. Table S2: summary of 75 recognized pyroptosis-related genes. Table S3: the primer sequences of CASP5, GBP1, GZMB, IL1B, and IRF1. Table S4: differential expression analysis of pyroptosis-related genes in the training set (active UC vs. inactive UC). Table S5: differential expression analysis of pyroptosis-related genes in GSE75214 (active UC vs. inactive UC). Table S6: standard weight of each gene in WGCNA. Table S7: logistic regression analysis for the key PRGs. Table S8: correlation of clinicopathologic characteristics and PR-Score in GSE111889. Table S9: correlation of clinicopathologic characteristics and PR-Score in GSE94648. Table S10: correlation of clinicopathologic characteristics and PR-Score in TCGA. Table S11: correlation of clinicopathologic characteristics and PR-Score in GSE39582. [file 7040113.f1.zip › Supplementary figures and legends.pdf]

Supplementary figure legends

Figure S1. The principal component analysis (PCA) of before **(a)** and after **(b)** batch correction of all samples in the training set.

Figure S2. Determination of soft-thresholding power in the co-expression network. (a) The scale-free fit index was analyzed for various soft-thresholding powers (β). **(b)** Analysis of the mean connectivity for various soft-thresholding powers. **(c)** Histogram of connectivity distribution when $\beta = 5$. **(d)** Checking the scale-free topology when $\beta = 5$.

Figure S3. (a) The correlation of the PR-Score and Mayo score in GSE94648. Using unsupervised consensus clustering, UC samples were reclassified into two clusters in the training set **(b)**, GSE75214 **(c)** and GSE94648 **(d)**. **(e)** Distribution of two clusters in active UC and inactive UC in GSE75214 (left) and GSE94648 (right). **(f)** UC samples of GSE111889 were classified into two clusters using unsupervised consensus clustering. UC, ulcerative colitis; ac, active; in, inactive

Figure S4. Representative IHC images showing the expressions of 5 PR-signature genes in normal, DSS-induced colitis, AOM/DSS-induced CAC tissues of mice.

Figure S1

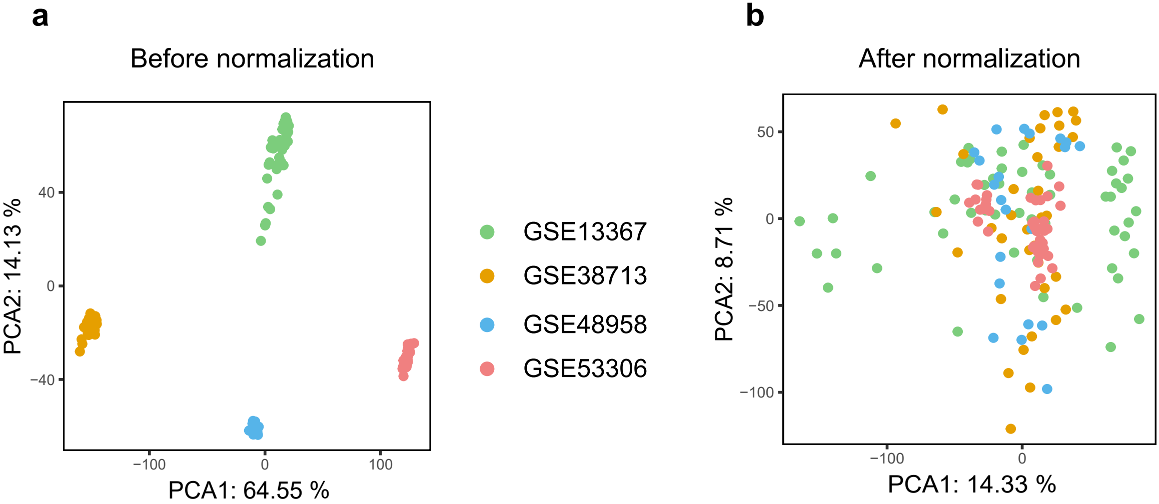
a
Before normalization
PCA2: 14.13 %
40
0
−40
−100
0
100
PCA1: 64.55 %
GSE13367
GSE38713
GSE48958
GSE53306
b
After normalization
PCA2: 8.71 %
50
0
−50
−100
−100
0
PCA1: 14.33 %

Figure S2

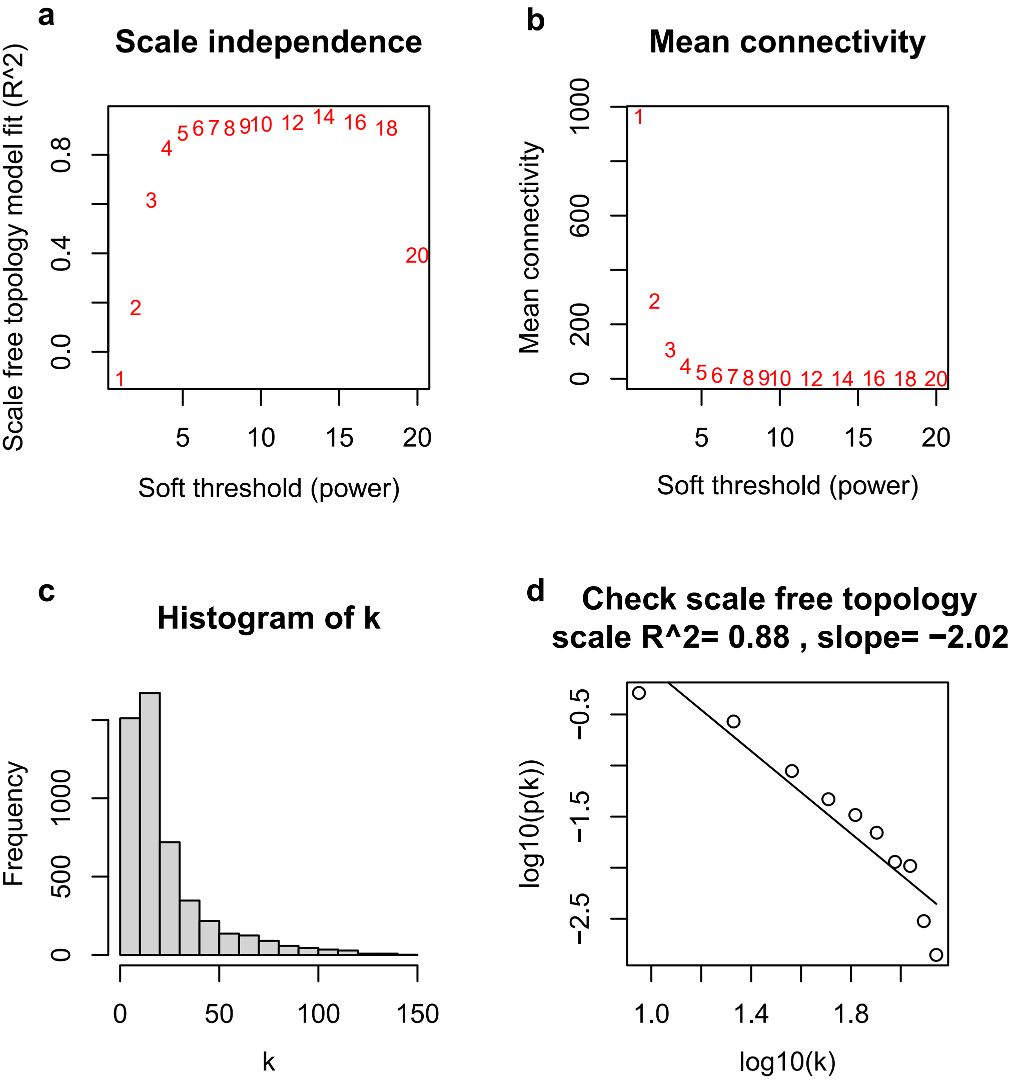
a
Scale independence
Scale free topology model fit (R^2)
Soft threshold (power)
b
Mean connectivity
Mean connectivity
Soft threshold (power)
c
Histogram of k
Frequency
k
d
Check scale free topology
scale R^2= 0.88 , slope= −2.02
log10(p(k))
log10(k)

Figure S3

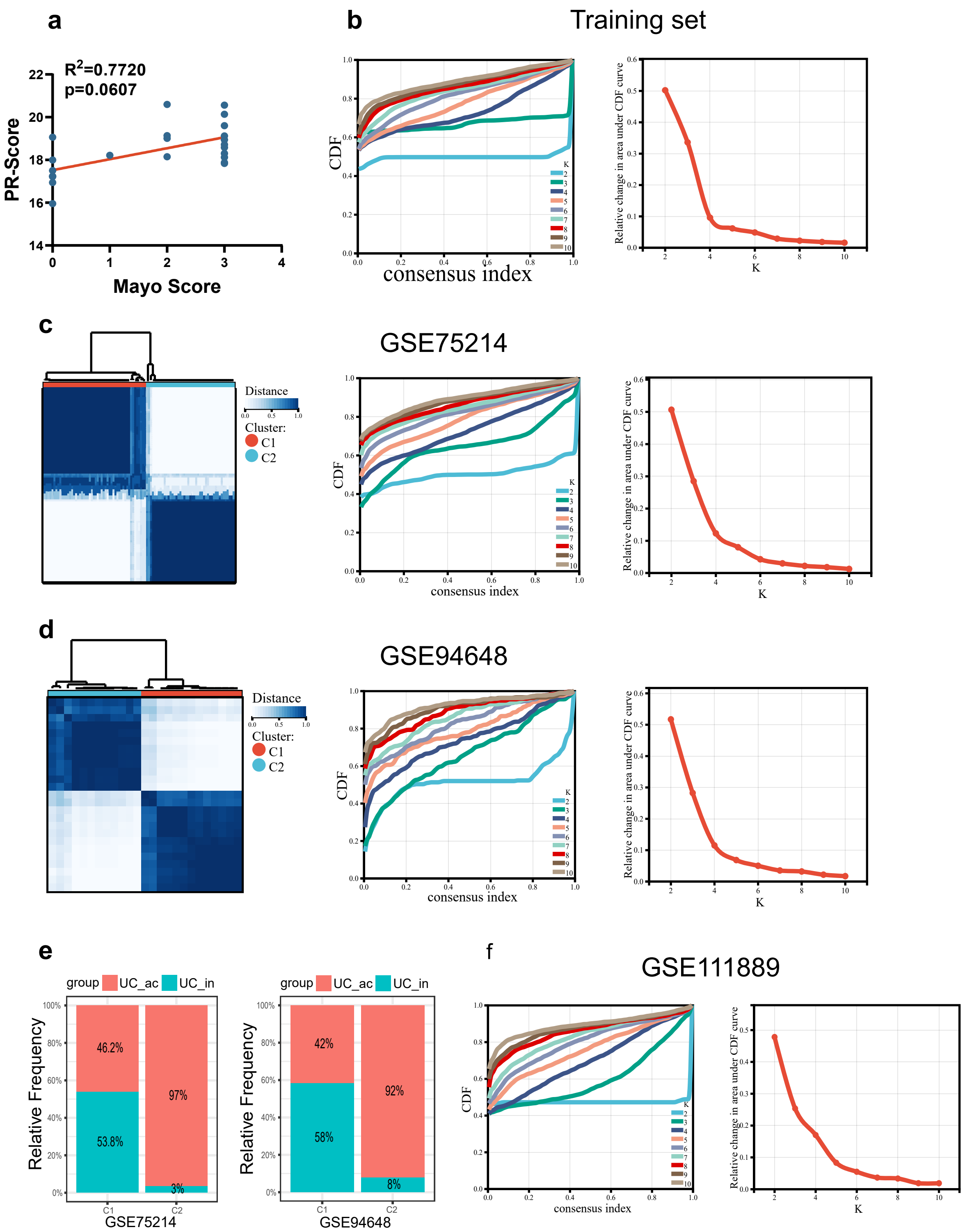
a
b
Training set
R^2=0.7720
p=0.0607
PR-Score
Mayo Score
CDF
consensus index
Relative change in area under CDF curve
K
c
GSE75214
Distance
Cluster:
C1
C2
d
GSE94648
e
group
UC_ac
UC_in
Relative Frequency
46.2%
53.8%
97%
3%
42%
58%
92%
8%
C1
C2
GSE75214
GSE94648
f
GSE111889

Figure S4

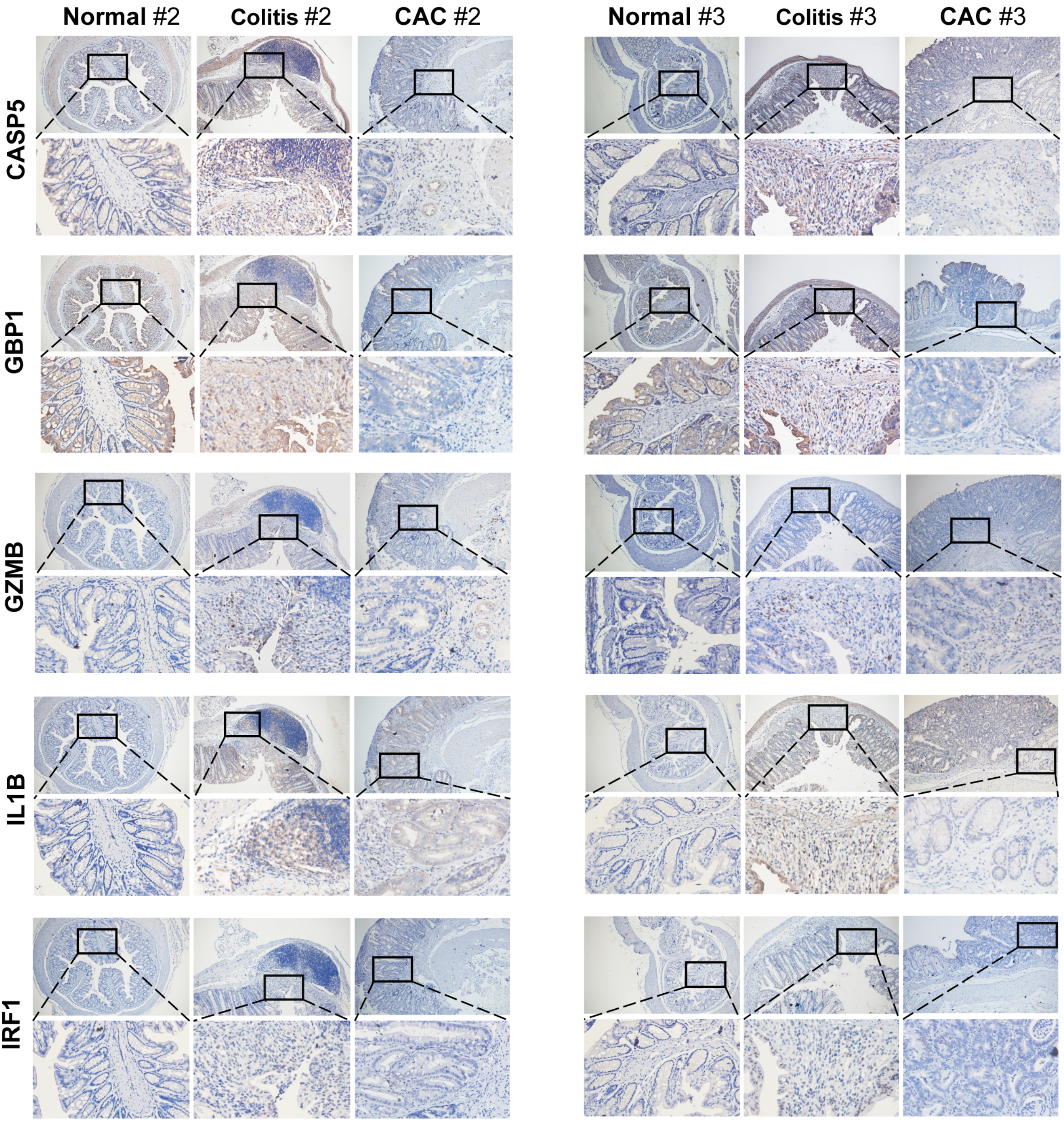

Normal #2
Colitis #2
CAC #2
Normal #3
Colitis #3
CAC #3
CASP5
GBP1
GZMB
IL1B
IRF1
