# Supplementary material for: Pyroptosis-Related Signature Predicts the Progression of Ulcerative Colitis and Colitis-Associated Colorectal Cancer as well as the Anti-TNF Therapeutic Response
Source: J Immunol Res. 2023 Jan 27;2023:7040113. doi: 10.1155/2023/7040113 (PMC9897931; doi:10.1155/2023/7040113)
Supplement: Supplementary Materials — Figure S1: the principal component analysis of before and after batch correction of all samples in the training set. Figure S2: determination of soft-thresholding power in the coexpression network. Figure S3: the correlation of the PR-Score and PR-clusters. Figure S4: representative IHC images showing the expressions of 5 PR-signature genes in normal, DSS-induced colitis, and AOM/DSS-induced CAC tissues of mice. Table S1: detailed information of all datasets and included samples. Table S2: summary of 75 recognized pyroptosis-related genes. Table S3: the primer sequences of CASP5, GBP1, GZMB, IL1B, and IRF1. Table S4: differential expression analysis of pyroptosis-related genes in the training set (active UC vs. inactive UC). Table S5: differential expression analysis of pyroptosis-related genes in GSE75214 (active UC vs. inactive UC). Table S6: standard weight of each gene in WGCNA. Table S7: logistic regression analysis for the key PRGs. Table S8: correlation of clinicopathologic characteristics and PR-Score in GSE111889. Table S9: correlation of clinicopathologic characteristics and PR-Score in GSE94648. Table S10: correlation of clinicopathologic characteristics and PR-Score in TCGA. Table S11: correlation of clinicopathologic characteristics and PR-Score in GSE39582. [file 7040113.f1.zip › Supplementary tables.pdf]

**Table S1. Detailed information of all data sets and included samples.**

| | Data set | Included samples |
|---|---|---|
| Merged training set | GSE13367 | 20 normal, 18 UC non-inflamed, 16 UC inflamed |
| | GSE38713 | 13 normal, 8 UC-remission, 15 UC-active |
| | GSE48958 | 8 normal, 6 UC-inactive, 7 UC-active |
| | GSE53306 | 12 normal, 12 UC-inactive, 16 UC-active |
| Validation set | GSE75214 | 22 normal, 23 UC-inactive, 74 UC-active |
| | GSE94648 | 8 UC-inactive, 17 UC-active |
| Clustering cohort | GSE111889 | 73 UC |
| Therapy cohort | GSE16879 | 48 UC patients treated with infliximab |
| | GSE23597 | 113 UC patients treated with infliximab |
| | GSE92415 | 162 UC patients treated with golimumab |
| UC-associated dysplasia/cancer set | GSE4183 | 8 normal, 15 IBD, 15 adenoma, 15 CRC |
| | GSE47908 | 20 left-sided coltis, 19 pancolitis, 6 UC-associated dysplasia |
| | GSE3629 | 53 UC-nonneoplastic mucosa, 68 CRC |
| CRC cohort | TCGA | 417 patients with survival information |
| | GSE39582 | 566 patients with survival information |

**Table S2: Summary of 75 recognized pyroptosis-related genes.**

| **Gene** | **Type** |
|---|---|
| AIM2 | pyroptosis |
| APIP | pyroptosis |
| BAK1 | pyroptosis |
| BAX | pyroptosis |
| CARD8 | pyroptosis |
| CASP1 | pyroptosis |
| CASP3 | pyroptosis |
| CASP4 | pyroptosis |
| CASP5 | pyroptosis |
| CASP6 | pyroptosis |
| CASP8 | pyroptosis |
| CASP9 | pyroptosis |
| CHMP2A | pyroptosis |
| CHMP2B | pyroptosis |
| CHMP4B | pyroptosis |
| CHMP4C | pyroptosis |

| | |
|---|---|
| CHMP6 | pyroptosis |
| CHMP7 | pyroptosis |
| CYCS | pyroptosis |
| DDX3X | pyroptosis |
| DDX58 | pyroptosis |
| DHX9 | pyroptosis |
| DIABLO | pyroptosis |
| DPP8 | pyroptosis |
| DPP9 | pyroptosis |
| EEF2K | pyroptosis |
| ELANE | pyroptosis |
| ELAVL1 | pyroptosis |
| FOXO3 | pyroptosis |
| GBP1 | pyroptosis |
| GJA1 | pyroptosis |
| GPX4 | pyroptosis |
| GSDMB | pyroptosis |
| GSDMC | pyroptosis |
| GSDMD | pyroptosis |
| GZMA | pyroptosis |
| GZMB | pyroptosis |
| HMGB1 | pyroptosis |
| IKBKB | pyroptosis |
| IL18 | pyroptosis |
| IL1A | pyroptosis |
| IL1B | pyroptosis |
| IL6 | pyroptosis |
| IRF1 | pyroptosis |
| IRF2 | pyroptosis |
| MALT1 | pyroptosis |
| MAP3K7 | pyroptosis |
| MAPK8 | pyroptosis |
| NAIP | pyroptosis |
| NFKB1 | pyroptosis |
| NLRC4 | pyroptosis |
| NLRP1 | pyroptosis |
| NLRP2 | pyroptosis |
| NLRP3 | pyroptosis |
| NLRP6 | pyroptosis |
| NLRP7 | pyroptosis |
| NLRP9 | pyroptosis |
| NOD1 | pyroptosis |
| NOD2 | pyroptosis |
| P2RX7 | pyroptosis |

| | |
|---|---|
| PELP1 | pyroptosis |
| PLCG1 | pyroptosis |
| PLK1 | pyroptosis |
| PRKACA | pyroptosis |
| PYCARD | pyroptosis |
| ROS1 | pyroptosis |
| SCGB3A2 | pyroptosis |
| STAT1 | pyroptosis |
| STK4 | pyroptosis |
| TET2 | pyroptosis |
| TIRAP | pyroptosis |
| TNF | pyroptosis |
| TP53 | pyroptosis |
| TP63 | pyroptosis |
| TREM2 | pyroptosis |

**Table S3 The primer sequences of the PR-signature genes.**

| | |
|---|---|
| GzmB-F | CCCTGGGAAAACACTCACACA |
| GzmB-R | GCACAACTCAATGGTACTGTCG |
| IRF1-F | ATGCCCATCACTCGGATGC |
| IRF1-R | CCCTGCTTTGTATCGGCCTG |
| IL-1β-F | CTCGCCAGTGAAATGATGGCT |
| IL-1β-R | GTCGGAGATTCGTAGCTGGAT |
| Casp5-F | GCAAGGAATGGGGCTCACTA |
| Casp5-R | CGTGCTGTCAGAGGACTTGT |
| GBP1-F | TATTGCCCACTATGAACAGCAGAT |
| GBP1-R | TAGCTGGGCCGCTAACTCC |
| GAPDH-Forward | CCCCAGCAAGAGCACAAGAG |
| GAPDH-Reverse | GCACAGGGTACTTTATTGATGGTAC |

**Table S4: Differential expression analysis of pyroptosis-related genes in training set (active UC vs inactive UC).**

| symbol | logFC | AveExpr | t | P.Value | adj.P.Val | B |
|---|---|---|---|---|---|---|
| IL1B | 2.314339 | 9.424312 | 8.50465 | 1.94E-13 | 1.45E-11 | 20.08097 |
| IRF1 | 1.168373 | 9.457879 | 7.7033 | 1.02E-11 | 3.82E-10 | 16.1769 |
| CASP1 | 0.979067 | 9.980213 | 7.37564 | 5.05E-11 | 1.26E-09 | 14.58348 |
| CASP5 | 1.445441 | 9.843297 | 6.88184 | 5.39E-10 | 1.01E-08 | 12.27779 |
| GZMB | 1.446873 | 7.543649 | 6.59211 | 2.11E-09 | 3.17E-08 | 10.93753 |
| GBP1 | 1.3017 | 9.659696 | 6.38389 | 5.58E-09 | 6.97E-08 | 9.988044 |
| CASP4 | 0.70361 | 8.367881 | 6.3069 | 7.96E-09 | 8.52E-08 | 9.640156 |

| | | | | | | |
|---|---|---|---|---|---|---|
| STAT1 | 0.710436 | 11.81226 | 5.56425 | 2.24E-07 | 2.10E-06 | 6.384567 |
| IL6 | 1.171211 | 5.917978 | 5.21094 | 1.02E-06 | 8.53E-06 | 4.909399 |
| AIM2 | 0.907281 | 7.492253 | 5.15798 | 1.28E-06 | 9.59E-06 | 4.692938 |
| MALT1 | 0.486698 | 7.739172 | 4.63535 | 1.09E-05 | 7.41E-05 | 2.628221 |
| PLK1 | 0.424711 | 7.165375 | 4.40494 | 2.68E-05 | 0.0001673 | 1.762616 |
| NAIP | 0.577279 | 6.530341 | 4.35699 | 3.22E-05 | 0.0001857 | 1.586162 |
| NLRP7 | 0.464496 | 5.652592 | 4.23492 | 5.12E-05 | 0.0002741 | 1.14288 |
| APIP | 0.492225 | 9.315593 | 3.93984 | 0.0001518 | 0.0007588 | 0.10802 |
| GSDMD | 0.256708 | 9.827353 | 3.88946 | 0.0001818 | 0.0008022 | -0.063273 |
| PRKACA | -0.2088 | 8.21782 | -3.89834 | 0.0001761 | 0.0008022 | -0.033187 |
| CARD8 | 0.661077 | 7.481506 | 3.78598 | 0.0002623 | 0.001093 | -0.410016 |
| NOD2 | 0.402796 | 6.169012 | 3.44008 | 0.000852 | 0.0033632 | -1.517368 |
| DDX58 | 0.548025 | 7.155197 | 3.33823 | 0.0011881 | 0.0044054 | -1.827628 |
| IL18 | 0.373093 | 9.934341 | 3.32662 | 0.0012335 | 0.0044054 | -1.862537 |
| FOXO3 | 0.269017 | 10.50738 | 3.21318 | 0.0017709 | 0.0058052 | -2.198398 |
| DPP8 | -0.26314 | 9.883378 | -3.2115 | 0.0017803 | 0.0058052 | -2.2033 |
| BAK1 | 0.208014 | 7.940209 | 3.15869 | 0.0021004 | 0.0065638 | -2.356362 |
| NLRC4 | 0.332327 | 5.205332 | 3.04745 | 0.0029576 | 0.00857 | -2.671977 |
| CYCS | -0.40019 | 11.66639 | -3.04597 | 0.0029709 | 0.00857 | -2.676113 |
| NOD1 | 0.239561 | 7.657563 | 3.02645 | 0.003152 | 0.0087557 | -2.730515 |
| TNF | 0.218982 | 5.436664 | 2.86167 | 0.0051401 | 0.0137682 | -3.178035 |
| PELP1 | -0.21898 | 8.392203 | -2.62124 | 0.0101392 | 0.0262221 | -3.792632 |
| IL1A | 0.252623 | 5.482898 | 2.36078 | 0.0201911 | 0.0295297 | -4.405159 |

**Table S5: Differential expression analysis of pyroptosis-related genes in GSE75214 (active UC vs inactive UC).**

| symbol | logFC | AveExpr | t | P.Value | adj.P.Val | B |
|---|---|---|---|---|---|---|
| GBP1 | 1.449041 | 10.38292 | 10.5925 | 6.66E-18 | 5.46E-16 | 30.14336 |
| CASP4 | 1.069865 | 8.264716 | 9.98152 | 1.39E-16 | 5.71E-15 | 27.12426 |
| STAT1 | 1.035259 | 10.63243 | 9.66405 | 6.79E-16 | 1.85E-14 | 25.55355 |
| NLRP7 | 0.49129 | 5.463552 | 9.15859 | 8.43E-15 | 1.73E-13 | 23.055 |
| CASP8 | 0.541682 | 8.157328 | 8.73493 | 6.93E-14 | 1.14E-12 | 20.96769 |
| IRF1 | 0.98716 | 9.614628 | 8.67976 | 9.11E-14 | 1.25E-12 | 20.69664 |
| CARD8 | 0.718574 | 7.574515 | 7.58224 | 2.00E-11 | 2.05E-10 | 15.365 |
| CASP1 | 1.012162 | 9.068115 | 7.4574 | 3.66E-11 | 2.73E-10 | 14.76856 |
| GZMB | 0.766974 | 7.04794 | 7.46609 | 3.51E-11 | 2.73E-10 | 14.81 |
| IL1A | 1.969402 | 6.72836 | 7.48413 | 3.21E-11 | 2.73E-10 | 14.89605 |
| IL1B | 2.43116 | 8.994075 | 7.43413 | 4.09E-11 | 2.80E-10 | 14.6577 |
| CASP5 | 1.516798 | 10.23524 | 7.06251 | 2.43E-10 | 1.53E-09 | 12.90049 |
| CYCS | -0.56357 | 8.934312 | -7.00479 | 3.20E-10 | 1.87E-09 | 12.63007 |
| CHMP6 | -0.3168 | 7.732322 | -6.9575 | 4.00E-10 | 2.19E-09 | 12.40905 |
| GJA1 | 1.365594 | 7.771706 | 6.86174 | 6.30E-10 | 3.23E-09 | 11.96304 |

| | | | | | | |
|---|---|---|---|---|---|---|
| NOD2 | 0.622231 | 6.540753 | 6.77934 | 9.29E-10 | 4.48E-09 | 11.58095 |
| MALT1 | 0.462782 | 7.873946 | 6.55711 | 2.63E-09 | 1.20E-08 | 10.55887 |
| AIM2 | 1.185252 | 6.195978 | 6.4556 | 4.20E-09 | 1.72E-08 | 10.09633 |
| CHMP4B | -0.24507 | 9.408534 | -6.21015 | 1.30E-08 | 5.07E-08 | 8.990058 |
| NLRP1 | 0.54752 | 6.849466 | 6.15777 | 1.65E-08 | 6.14E-08 | 8.756321 |
| EEF2K | -0.30334 | 7.838162 | -5.89021 | 5.49E-08 | 1.96E-07 | 7.576361 |
| GSDMC | 0.561349 | 4.788263 | 5.77651 | 9.10E-08 | 3.11E-07 | 7.082362 |
| IL6 | 2.034506 | 6.583625 | 5.72486 | 1.14E-07 | 3.75E-07 | 6.859514 |
| DDX58 | 0.58975 | 7.836988 | 5.63497 | 1.70E-07 | 5.15E-07 | 6.474025 |
| PLK1 | 0.645181 | 8.695784 | 5.64249 | 1.64E-07 | 5.15E-07 | 6.506165 |
| DIABLO | -0.25732 | 7.530863 | -5.47712 | 3.37E-07 | 9.87E-07 | 5.804673 |
| GPX4 | -0.39082 | 9.801584 | -5.22519 | 9.88E-07 | 2.79E-06 | 4.75752 |
| P2RX7 | 0.590875 | 6.222397 | 5.1761 | 1.22E-06 | 3.32E-06 | 4.556644 |
| PRKACA | -0.17472 | 8.648669 | -4.9323 | 3.34E-06 | 8.84E-06 | 3.575353 |
| NLRC4 | 0.672343 | 5.435607 | 4.82438 | 5.18E-06 | 1.33E-05 | 3.150028 |
| PELP1 | -0.18422 | 7.278771 | -4.71692 | 7.99E-06 | 1.98E-05 | 2.732221 |
| GSDMD | 0.298878 | 7.654334 | 4.53003 | 1.67E-05 | 4.03E-05 | 2.019824 |
| NLRP3 | 0.778402 | 6.379648 | 4.49139 | 1.94E-05 | 4.55E-05 | 1.874846 |
| DPP9 | 0.213402 | 8.409506 | 4.42313 | 2.53E-05 | 5.77E-05 | 1.620758 |
| TNF | 0.502555 | 6.717923 | 4.41264 | 2.64E-05 | 5.84E-05 | 1.581938 |
| STK4 | 0.348643 | 9.165672 | 4.35873 | 3.24E-05 | 7.00E-05 | 1.383401 |
| IRF2 | 0.183885 | 9.257544 | 3.96432 | 0.0001404 | 0.0002877 | -0.01755 |
| CHMP2A | -0.21294 | 10.83294 | -3.95472 | 0.0001453 | 0.0002906 | -0.050472 |
| HMGB1 | 0.200035 | 10.6057 | 3.90555 | 0.0001733 | 0.0003384 | -0.218216 |
| TP53 | -0.35454 | 9.189183 | -3.86682 | 0.000199 | 0.0003794 | -0.349249 |
| CASP6 | -0.44923 | 7.774471 | -3.73757 | 0.0003133 | 0.0005839 | -0.7796 |
| NAIP | 0.520264 | 8.637826 | 3.63148 | 0.0004514 | 0.0008226 | -1.124634 |
| PYCARD | -0.19145 | 8.83104 | -3.45825 | 0.0008075 | 0.0014394 | -1.671625 |
| CASP3 | 0.194319 | 8.455423 | 3.43074 | 0.0008841 | 0.0015424 | -1.756573 |
| NLRP6 | 0.137207 | 6.822703 | 3.24309 | 0.0016194 | 0.00271 | -2.321716 |
| TET2 | 0.183959 | 8.531719 | 2.94056 | 0.0040915 | 0.0067101 | -3.178285 |
| DPP8 | -0.15142 | 8.342707 | -2.93318 | 0.0041819 | 0.0067238 | -3.198324 |
| TIRAP | -0.13661 | 6.879001 | -2.92188 | 0.0043238 | 0.0068183 | -3.228915 |
| CASP9 | -0.17308 | 8.086877 | -2.87985 | 0.0048911 | 0.0075674 | -3.341789 |
| ROS1 | -0.22827 | 4.675049 | -2.68371 | 0.0085552 | 0.0129912 | -3.850198 |
| MAPK8 | 0.13343 | 8.656983 | 2.5236 | 0.0132317 | 0.0197273 | -4.24222 |
| DDX3X | 0.176436 | 9.193896 | 2.4071 | 0.0179617 | 0.026301 | -4.514165 |
| IL18 | 0.43069 | 8.883834 | 2.34378 | 0.0211189 | 0.0303816 | -4.657187 |
| GZMA | 0.238318 | 6.355486 | 2.2913 | 0.0240976 | 0.0340691 | -4.773133 |
| ELAVL1 | -0.09542 | 8.288474 | -2.22438 | 0.0284289 | 0.0395113 | -4.91757 |

**Table S6: Standard weight of each gene in WGCNA.**

| GeneName | p.Standard | q.Standard | cor.Standard | Z.Standard |
|---|---|---|---|---|
| PI3 | 0 | 0 | 0.75381 | 11.18138 |
| SLC6A14 | 0 | 0 | 0.73534 | 10.575867 |
| LCN2 | 0 | 0 | 0.73438 | 10.545705 |
| DUOX2 | 0 | 0 | 0.72558 | 10.277138 |
| S100A8 | 0 | 0 | 0.72514 | 10.263873 |
| DUOXA2 | 0 | 0 | 0.7245 | 10.244905 |
| TCN1 | 0 | 0 | 0.71789 | 10.051157 |
| PFKFB3 | 2.22E-16 | 3.27E-14 | 0.71276 | 9.9044207 |
| CXCL1 | 2.22E-16 | 3.27E-14 | 0.71265 | 9.9014548 |
| GNA15 | 4.44E-16 | 5.45E-14 | 0.71044 | 9.8393966 |
| FKBP11 | 6.66E-16 | 7.55E-14 | 0.70665 | 9.7343124 |
| LPCAT1 | 2.22E-15 | 2.34E-13 | 0.69695 | 9.4726111 |
| NCF2 | 3.11E-15 | 3.05E-13 | 0.69437 | 9.4047021 |
| S100A9 | 6.22E-15 | 5.72E-13 | 0.68872 | 9.2587899 |
| TNIP3 | 9.10E-15 | 7.89E-13 | 0.68587 | 9.1861469 |
| LYN | 1.87E-14 | 1.37E-12 | 0.67998 | 9.0388496 |
| KYNU | 2.09E-14 | 1.46E-12 | 0.67914 | 9.0182267 |
| PDZK1IP1 | 2.98E-14 | 1.95E-12 | 0.67617 | 8.9454963 |
| BACE2 | 3.04E-14 | 1.95E-12 | 0.67594 | 8.9399215 |
| IL1B | 1.51E-13 | 8.14E-12 | 0.6622 | 8.6134009 |
| C4BPA | 1.55E-13 | 8.14E-12 | 0.66199 | 8.6085953 |
| SLC7A5 | 2.32E-13 | 1.18E-11 | 0.65839 | 8.5257698 |
| MMP7 | 2.63E-13 | 1.29E-11 | 0.65727 | 8.5001657 |
| IFITM2 | 3.51E-13 | 1.67E-11 | 0.65465 | 8.4409725 |
| PLAUR | 3.91E-13 | 1.80E-11 | 0.65367 | 8.4189118 |
| DDIT4 | 4.45E-13 | 1.99E-11 | 0.65251 | 8.3927957 |
| IFITM1 | 5.40E-13 | 2.27E-11 | 0.65072 | 8.3528878 |
| CD55 | 6.42E-13 | 2.63E-11 | 0.64913 | 8.3175378 |
| IFITM3 | 6.66E-13 | 2.65E-11 | 0.6488 | 8.3100991 |
| BIRC3 | 7.69E-13 | 2.97E-11 | 0.64746 | 8.2806259 |
| FAM49A | 9.52E-13 | 3.42E-11 | 0.64547 | 8.2368417 |
| APOL1 | 1.01E-12 | 3.46E-11 | 0.64495 | 8.2256203 |
| CHI3L1 | 1.01E-12 | 3.46E-11 | 0.64488 | 8.2240463 |
| MNDA | 1.03E-12 | 3.46E-11 | 0.64471 | 8.2202382 |
| CARD16 | 1.61E-12 | 5.04E-11 | 0.6405 | 8.1291651 |
| SAMD9L | 2.10E-12 | 6.45E-11 | 0.63794 | 8.0741723 |
| ADM | 2.55E-12 | 7.62E-11 | 0.63604 | 8.0338663 |
| PARP9 | 2.59E-12 | 7.62E-11 | 0.63592 | 8.0313811 |
| RAC2 | 3.42E-12 | 9.87E-11 | 0.6332 | 7.9738904 |
| ANXA1 | 3.81E-12 | 1.08E-10 | 0.63213 | 7.9514845 |
| G0S2 | 4.16E-12 | 1.16E-10 | 0.63126 | 7.9331385 |
| IRF1 | 4.27E-12 | 1.17E-10 | 0.631 | 7.9276838 |
| SLC2A3 | 4.84E-12 | 1.28E-10 | 0.62977 | 7.9020461 |

| | | | | |
|---|---|---|---|---|
| PIM2 | 4.88E-12 | 1.28E-10 | 0.62968 | 7.9003419 |
| VNN2 | 5.08E-12 | 1.31E-10 | 0.62928 | 7.8919301 |
| CD44 | 5.83E-12 | 1.48E-10 | 0.6279 | 7.8633776 |
| MMP12 | 6.70E-12 | 1.67E-10 | 0.62652 | 7.8348781 |
| MAP3K8 | 8.43E-12 | 2.07E-10 | 0.6242 | 7.7873178 |
| GPX8 | 9.44E-12 | 2.28E-10 | 0.62305 | 7.7637907 |
| ZBP1 | 9.93E-12 | 2.34E-10 | 0.62253 | 7.7532755 |
| SAA1 | 9.99E-12 | 2.34E-10 | 0.62247 | 7.7519625 |
| REG1B | 1.03E-11 | 2.37E-10 | 0.62214 | 7.745368 |
| PROK2 | 1.08E-11 | 2.44E-10 | 0.6217 | 7.7363238 |
| IDO1 | 1.31E-11 | 2.93E-10 | 0.61968 | 7.6955908 |
| SORD | 1.54E-11 | 3.39E-10 | 0.61801 | 7.6619668 |
| AQP9 | 1.70E-11 | 3.63E-10 | 0.61701 | 7.6418897 |
| ASPHD2 | 1.82E-11 | 3.83E-10 | 0.61628 | 7.6274138 |
| SNCAIP | 1.87E-11 | 3.87E-10 | 0.61603 | 7.6223647 |
| REG1A | 1.98E-11 | 4.06E-10 | 0.61539 | 7.6096867 |
| IFNAR2 | 2.43E-11 | 4.91E-10 | 0.61324 | 7.5669296 |
| MMP10 | 2.57E-11 | 5.04E-10 | 0.61268 | 7.5559159 |
| ERO1L | 2.76E-11 | 5.35E-10 | 0.61192 | 7.5409016 |
| C4BPB | 4.24E-11 | 8.11E-10 | 0.60733 | 7.451128 |
| GABRP | 4.30E-11 | 8.13E-10 | 0.60717 | 7.4480006 |
| PDE4B | 4.43E-11 | 8.26E-10 | 0.60686 | 7.442003 |
| FUT8 | 4.85E-11 | 8.86E-10 | 0.60588 | 7.4228981 |
| VSNL1 | 4.87E-11 | 8.86E-10 | 0.60584 | 7.4221641 |
| MMP3 | 5.14E-11 | 9.23E-10 | 0.60525 | 7.4108471 |
| WARS | 5.74E-11 | 1.02E-09 | 0.60405 | 7.3877325 |
| ETV7 | 5.79E-11 | 1.02E-09 | 0.60396 | 7.385862 |
| TAP1 | 6.19E-11 | 1.07E-09 | 0.60324 | 7.3720935 |
| LEPRE1 | 6.98E-11 | 1.20E-09 | 0.60192 | 7.3467954 |
| ACPP | 7.32E-11 | 1.24E-09 | 0.6014 | 7.3368564 |
| CASP1 | 7.79E-11 | 1.29E-09 | 0.60071 | 7.3235823 |
| SLA | 1.23E-10 | 1.97E-09 | 0.59566 | 7.2280593 |
| BCL2A1 | 1.37E-10 | 2.13E-09 | 0.59441 | 7.2044724 |
| HIF1A | 1.59E-10 | 2.44E-09 | 0.59275 | 7.1734563 |
| PPP1R16B | 1.74E-10 | 2.64E-09 | 0.59174 | 7.1545966 |
| RPN2 | 1.88E-10 | 2.83E-09 | 0.59083 | 7.1377314 |
| SELL | 1.94E-10 | 2.86E-09 | 0.59052 | 7.1319578 |
| PCSK1 | 1.94E-10 | 2.86E-09 | 0.59049 | 7.1315033 |
| PSMB9 | 2.22E-10 | 3.21E-09 | 0.58894 | 7.1028063 |
| CFI | 2.29E-10 | 3.27E-09 | 0.5886 | 7.0965242 |
| PMAIP1 | 2.70E-10 | 3.71E-09 | 0.58672 | 7.0618816 |
| PDK1 | 2.97E-10 | 4.06E-09 | 0.58559 | 7.0411235 |
| DMBT1 | 3.14E-10 | 4.24E-09 | 0.58496 | 7.0296301 |
| CCDC109B | 3.39E-10 | 4.54E-09 | 0.58405 | 7.0130872 |

| | | | | |
|---|---|---|---|---|
| LAX1 | 3.52E-10 | 4.63E-09 | 0.58364 | 7.005594 |
| CD38 | 3.55E-10 | 4.63E-09 | 0.58352 | 7.003479 |
| GPX7 | 3.70E-10 | 4.78E-09 | 0.58303 | 6.9945396 |
| TGM2 | 4.28E-10 | 5.43E-09 | 0.58134 | 6.9638653 |
| ENTPD1 | 4.53E-10 | 5.71E-09 | 0.58066 | 6.951562 |
| BASP1 | 4.64E-10 | 5.79E-09 | 0.58038 | 6.9465782 |
| NOS2 | 4.96E-10 | 6.04E-09 | 0.57959 | 6.9322442 |
| BCL6 | 5.37E-10 | 6.43E-09 | 0.57865 | 6.9153456 |
| UBE2L6 | 6.28E-10 | 7.23E-09 | 0.57679 | 6.8820769 |
| SPINK4 | 7.42E-10 | 8.41E-09 | 0.57479 | 6.8463375 |
| SH3BP5 | 8.40E-10 | 9.44E-09 | 0.5733 | 6.8199009 |
| CYB5R2 | 9.49E-10 | 1.06E-08 | 0.57182 | 6.7937095 |
| SAMSN1 | 1.11E-09 | 1.21E-08 | 0.56994 | 6.7605422 |
| ISG20 | 1.17E-09 | 1.26E-08 | 0.56925 | 6.7484804 |
| TCIRG1 | 1.23E-09 | 1.30E-08 | 0.56864 | 6.7378725 |
| IL15RA | 1.31E-09 | 1.37E-08 | 0.56785 | 6.7239589 |
| CASP5 | 1.36E-09 | 1.42E-08 | 0.56738 | 6.715748 |
| CCR1 | 1.41E-09 | 1.46E-08 | 0.56693 | 6.7079744 |
| CXCL6 | 1.44E-09 | 1.47E-08 | 0.56674 | 6.7045789 |
| CDC25B | 1.49E-09 | 1.52E-08 | 0.56627 | 6.6964489 |
| STOM | 1.67E-09 | 1.69E-08 | 0.56487 | 6.6720559 |
| EVI2B | 1.88E-09 | 1.88E-08 | 0.5634 | 6.6467145 |
| NAMPT | 2.05E-09 | 2.02E-08 | 0.56232 | 6.6280142 |
| CADPS2 | 2.22E-09 | 2.15E-08 | 0.5613 | 6.6104716 |
| CYP4X1 | 2.25E-09 | 2.17E-08 | 0.56115 | 6.607803 |
| SLC5A1 | 2.40E-09 | 2.30E-08 | 0.56034 | 6.5938719 |
| ZNFX1 | 2.46E-09 | 2.34E-08 | 0.56004 | 6.5888443 |
| GZMB | 2.55E-09 | 2.39E-08 | 0.55959 | 6.5811239 |
| MMP1 | 2.84E-09 | 2.63E-08 | 0.5582 | 6.5573602 |
| FAM46C | 2.92E-09 | 2.69E-08 | 0.55786 | 6.5515706 |
| CRELD2 | 3.00E-09 | 2.73E-08 | 0.55752 | 6.5458067 |
| STS | 3.38E-09 | 3.02E-08 | 0.55598 | 6.5194778 |
| OAS2 | 3.49E-09 | 3.05E-08 | 0.55558 | 6.5128449 |
| CEBPD | 3.49E-09 | 3.05E-08 | 0.55556 | 6.5125083 |
| GBP1 | 3.70E-09 | 3.21E-08 | 0.55482 | 6.4998142 |
| GALNT2 | 4.13E-09 | 3.55E-08 | 0.55341 | 6.4760514 |
| IFI6 | 4.15E-09 | 3.55E-08 | 0.55337 | 6.4753084 |
| PARP14 | 4.25E-09 | 3.62E-08 | 0.55303 | 6.4697171 |
| WDR54 | 4.59E-09 | 3.89E-08 | 0.55203 | 6.4528727 |
| FOXQ1 | 4.64E-09 | 3.90E-08 | 0.5519 | 6.4506852 |
| ASS1 | 4.87E-09 | 4.03E-08 | 0.55127 | 6.4400921 |
| ITGB2 | 5.07E-09 | 4.17E-08 | 0.55077 | 6.4316062 |

**Table S7: Logistic regression analysis for the key PRGs.**

Training set

| indepentvar | B | SE | z | p | OR[95%CI] |
|---|---|---|---|---|---|
| CASP5 | 1.307 | 0.281 | 4.658 | <0.0001 | 3.694[2.132,6.402] |
| GBP1 | 1.273 | 0.277 | 4.589 | <0.0001 | 3.572[2.074,6.153] |
| GZMB | 1.209 | 0.257 | 4.698 | <0.0001 | 3.349[2.023,5.545] |
| IL1B | 1.34 | 0.268 | 5.001 | <0.0001 | 3.821[2.259,6.461] |
| IRF1 | 2.171 | 0.439 | 4.946 | <0.0001 | 8.765[3.708,20.717] |

GSE75214

| indepentvar | B | SE | z | p | OR[95%CI] |
|---|---|---|---|---|---|
| CASP5 | 1.601 | 0.425 | 14.189 | <0.0001 | 4.957[2.155,11.400] |
| GBP1 | 4.901 | 1.552 | 9.976 | <0.0001 | 134.484[6.42,281.05] |
| GZMB | 5.146 | 1.604 | 10.298 | <0.0001 | 171.810[7.41,398.15] |
| IL1B | 1.524 | 0.439 | 12.072 | <0.0001 | 4.592[1.943,10.850] |
| IRF1 | 2.126 | 1.167 | 12.504 | <0.0001 | 61.93[6.29,109.66] |

**Table S8. Correlation of clinicopathologic characteristics and PR-Score in GSE111889.**

| Characteristics | N (%) | PR-Score | | *P* |
|---|---|---|---|---|
| | | High | Low | Chi squared test |
| **Total cases** | 73 (100%) | 37 | 36 | |
| **Gender** | | | | 0.524 |
| Male | 27 (36.99%) | 15 | 12 | |
| Female | 46 (63.01%) | 22 | 24 | |
| **Biopsy location** | | | | 0.011 |
| Ileum | 24 (32.88%) | 5 | 19 | |
| Cecum | 1 (1.37%) | 0 | 1 | |
| Right-side | 3 (4.11%) | 2 | 1 | |
| Transverse colon | 5 (6.85%) | 2 | 3 | |
| Left-side | 4 (5.48%) | 3 | 1 | |
| Sigmoid colon | 9 (21.33%) | 6 | 3 | |
| Rectum | 26 (35.62%) | 19 | 7 | |

Note: The marked in red are statistically significant.

**Table S9. Correlation of clinicopathologic characteristics and PR-Score in GSE94648.**

| Characteristics | N (%) | PR-Score | | *P* |
|---|---|---|---|---|
| | | High | Low | Chi squared test |
| **Total cases** | 25 (100%) | 13 | 12 | |
| **Type** | | | | 0.007 |
| Active | 17 (68%) | 12 | 5 | |
| Inactive | 8 (32%) | 1 | 7 | |
| **Disease extension** | | | | 0.428 |
| Left-side colitis | 15 (60%) | 8 | 7 | |
| Pancolitis | 6 (24%) | 4 | 2 | |
| Procotilis | 4 (16%) | 1 | 3 | |
| **Mayo score** | | | | 0.007 |
| 0 | 8 (32%) | 1 | 7 | |
| ≥1 | 17 (68%) | 12 | 5 | |
| **Time of disease (years)** | | | | 0.546 |
| ≤5 | 6 (24%) | 4 | 2 | |
| ≤10 | 3 (12%) | 2 | 1 | |
| >10 | 16 (64%) | 7 | 8 | |

Note: Those marked in red are statistically significant.

**Table S10. Correlation of clinicopathologic characteristics and PR-Score in TCGA.**

| Characteristics | N (%) | PR-Score | *P* |
|---|---|---|---|

| | | High | Low | Chi squared test |
|---|---|---|---|---|
| **Total cases** | 417 (100%) | 208 | 209 | |
| **Gender** | | | | 0.655 |
| Male | 226 (54.20%) | 115 | 111 | |
| Female | 191 (45.80%) | 93 | 98 | |
| **Stage** | | | | <0.0001 |
| I | 70 (16.79%) | 43 | 27 | |
| II | 158 (37.89%) | 93 | 65 | |
| III | 119 (28.54%) | 49 | 70 | |
| IV | 59 (14.15%) | 17 | 42 | |
| **Lymphatic invasion** | | | | 0.042 |
| No | 230 (55.16%) | 128 | 102 | |
| Yes | 147 (35.25%) | 66 | 81 | |
| **Kras mutation** | | | | 0.023 |
| No | 23 (5.52%) | 7 | 16 | |
| Yes | 23 (5.52%) | 1 | 21 | |

Note: Those marked in red are statistically significant.

**Table S11. Correlation of clinicopathologic characteristics and PR-Score in GSE39582.**

| Characteristics | N (%) | PR-Score | | *P* |
|---|---|---|---|---|
| | | High | Low | Chi squared test |
| **Total cases** | 566 (100%) | 283 | 283 | |
| **Gender** | | | | 0.177 |
| Male | 310 (54.77%) | 163 | 147 | |
| Female | 256 (45.33%) | 120 | 136 | |
| **Stage** | | | | 0.013 |
| **0** | 4 (0.71%) | 3 | 1 | |
| I | 33 (5.83%) | 22 | 11 | |
| II | 264 (46.64%) | 140 | 124 | |
| III | 205 (36.22%) | 98 | 107 | |
| IV | 60 (10.60%) | 20 | 40 | |
| **Tumor location** | | | | 0.001 |
| Distal | 342 (60.42%) | 152 | 190 | |
| Proximal | 224 (39.58%) | 131 | 93 | |
| **Kras mutation** | | | | 0.052 |
| No | 328 (57.96%) | 176 | 152 | |
| Yes | 217 (42.04%) | 98 | 119 | |
| **Cit molecularsubtype** | | | | <0.0001 |
| C1 | 116 (20.49%) | 48 | 68 | |
| C2 | 104 (18.37%) | 92 | 12 | |
| C3 | 75 (13.25%) | 33 | 42 | |
| C4 | 59 (10.42%) | 27 | 32 | |
| C5 | 152 (26.86%) | 58 | 94 | |
| C6 | 60 (10.60%) | 25 | 35 | |

Note: Those marked in red are statistically significant.
